# The amino-terminus of the hepatitis C virus (HCV) p7 viroporin and its cleavage from glycoprotein E2-p7 precursor determine specific infectivity and secretion levels of HCV particle types

**DOI:** 10.1371/journal.ppat.1006774

**Published:** 2017-12-18

**Authors:** Solène Denolly, Chloé Mialon, Thomas Bourlet, Fouzia Amirache, François Penin, Brett Lindenbach, Bertrand Boson, François-Loïc Cosset

**Affiliations:** 1 CIRI–International Center for Infectiology Research, Team EVIR, Inserm, U1111, Université Claude Bernard Lyon 1, CNRS, UMR5308, Ecole Normale Supérieure de Lyon, Univ Lyon, Lyon, France; 2 GIMAP, EA 3064, Faculté de Médecine, Université de Saint-Etienne, Univ Lyon, Saint Etienne, France; 3 IBCP—Institut de Biologie et Chimie des Protéines, MMSB, UMR 5086, CNRS, Univ Lyon, Lyon, France; 4 Department of Microbial Pathogenesis, Yale School of Medicine, New Haven, CT, United States of America; University of California, San Diego, UNITED STATES

## Abstract

Viroporins are small transmembrane proteins with ion channel activities modulating properties of intracellular membranes that have diverse proviral functions. Hepatitis C virus (HCV) encodes a viroporin, p7, acting during assembly, envelopment and secretion of viral particles (VP). HCV p7 is released from the viral polyprotein through cleavage at E2-p7 and p7-NS2 junctions by signal peptidase, but also exists as an E2p7 precursor, of poorly defined properties. Here, we found that ectopic p7 expression in HCVcc-infected cells reduced secretion of particle-associated E2 glycoproteins. Using biochemical assays, we show that p7 dose-dependently slows down the ER-to-Golgi traffic, leading to intracellular retention of E2, which suggested that timely E2p7 cleavage and p7 liberation are critical events to control E2 levels. By studying HCV mutants with accelerated E2p7 processing, we demonstrate that E2p7 cleavage controls E2 intracellular expression and secretion levels of nucleocapsid-free subviral particles and infectious virions. In addition, our imaging data reveal that, following p7 liberation, the amino-terminus of p7 is exposed towards the cytosol and coordinates the encounter between NS5A and NS2-based assembly sites loaded with E1E2 glycoproteins, which subsequently leads to nucleocapsid envelopment. We identify punctual mutants at p7 membrane interface that, by abrogating NS2/NS5A interaction, are defective for transmission of infectivity owing to decreased secretion of core and RNA and to increased secretion of non/partially-enveloped particles. Altogether, our results indicate that the retarded E2p7 precursor cleavage is essential to regulate the intracellular and secreted levels of E2 through p7-mediated modulation of the cell secretory pathway and to unmask critical novel assembly functions located at p7 amino-terminus.

## Author summary

Viroporins are small transmembrane viral proteins with ion channel activities modulating properties of intracellular membranes, which impacts several fundamental biological processes such as trafficking, ion fluxes as well as connections and exchanges between organelles. Hepatitis C virus (HCV) encodes a viroporin, p7, acting during assembly, envelopment and secretion of viral particles. HCV p7 is produced by cleavage from the HCV polyprotein but also exists as an E2p7 precursor, of poorly defined properties. In this study, we have explored how the retarded cleavage between E2 glycoprotein and p7 viroporin could regulate their functions associated to virion assembly and/or perturbation of cellular membrane processes. Specifically, we demonstrate that p7 is able to regulate the cell secretory pathway, which induces the intracellular retention of HCV glycoproteins and favors assembly of HCV particles. Our study also identifies a novel assembly function located at p7 amino-terminus that is unmasked through E2p7-regulated processing and that controls the infectivity of different types of released viral particles. Altogether, our results underscore a critical post-translational control of assembly and secretion of HCV particles that governs their specific infectivity.

## Introduction

Hepatitis C virus (HCV) infection is a major cause of chronic liver diseases worldwide. With 180 million people persistently infected, chronic HCV infection induces liver diseases such as liver cirrhosis and hepatocellular carcinoma. Although new direct antiviral agents are now able to eradicate the virus in most patients, no protective vaccine currently exists against HCV and it remains major challenges in basic, translational and clinical research [1, 2].

HCV is a plus-strand RNA enveloped virus. Its genome is translated as a single polyprotein that is processed by cellular and viral proteases in 10 mature viral proteins [3] consisting of: i) an assembly module (core-E1-E2-p7-NS2) encompassing the capsid protein (core) as well as the E1 and E2 surface glycoproteins that are incorporated in viral particles, and the p7 and NS2 proteins that support virion assembly, and ii) a replication module encompassing the nonstructural proteins NS3, NS4A, NS4B, NS5A and NS5B that are sufficient to support viral RNA replication but that also contribute to virion production through ill-defined processes. As HCV proteins arise from a shared polyprotein, several post-translational modifications control their expression rates within infected cells. In addition, at least three precursors, *i*.*e*., tandem proteins with delayed cleavage, are also detected and may implement functions different than their cognate individual proteins. They consist of immature core protein, associated to the D3 trans-membrane peptide, whose removal allows core targeting to lipid droplets (LDs) [4, 5]; NS4B-5A, which promotes the formation of replication vesicles [6]; and E2p7 [7–11], whose properties are explored in this report.

Assembly of viral particles occurs at endoplasmic reticulum (ER)-derived membranes in close proximity to LDs and virus replication complexes [12], with NS2 and p7 being key players in gathering virion components [13–17]. Particularly, NS2 associates with E1E2 glycoproteins and NS3 as well as with NS5A, which interacts with HCV RNA and core [18–20] and promotes genome encapsidation. Virion assembly begins with the formation of a nucleocapsid, formed by core/RNA complex, and is coupled with its envelopment and acquisition of the E1E2 glycoproteins as well as lipids and apolipoproteins [18, 21]. The pathway of secretion of virions remains to be elucidated and may occur through a non-canonical route [22, 23]. Of note, HCV produces different types of particles in addition to infectious virions, including nucleocapsid-free subviral particles [24, 25], E2-containing exosome vesicles [26, 27], naked nucleocapsids [28], and a range of more or less lipidated infectious viral particles [29]; yet, the regulation of their production is poorly understood.

The p7 protein is a small, 63 amino-acid-long protein, consisting of a “hairpin-like” topology involving three helices inducing two trans-membrane segments connected by a hydrophilic, positively-charged cytosolic loop [30–32], though alternative folds and topologies have been proposed [33–35], *e*.*g*., with the p7 C-terminus exposed to the cytosol [33]. As it is able to form an ion channel in either hexameric or heptameric form [30, 35, 36] exhibiting a funnel- or flower-like shape [35, 37], it was classified as a viroporin, like M2 of influenza virus [38]. Importantly, p7 is dispensable for replication but essential for both assembly and secretion of infectious particles [11]. First, p7 modulates the formation of NS2 complexes with E2, NS3 and NS5A [9, 13, 14, 16, 39], allowing clustering of assembly components and regulation of early assembly events. Second, p7 allows, in concert with NS2, the regulation of core localization at lipid droplets *vs*. ER-derived membranes [17], from where viral particles are released in the secretory pathway. Third, p7 modulates the envelopment of nascent virions [40]. Fourth, p7 may regulate the pH of some intracellular compartments, which could be essential for the protection and secretion of infectious particles [41, 42].

A recent study indicated that residues of the first helix of p7 that are predicted to point toward the channel pore are important for assembly [10]. Noteworthy, viroporin ion channel activities modulate properties of intracellular membranes and, thereby, impacts several fundamental biological processes such as trafficking, ion fluxes as well as connections and exchanges between organelles [38, 43]. While several biophysical studies showed that p7 can change ionic gradients in reconstituted membrane assays *in vitro* [30, 44–47], few reports have addressed the relevance of such properties *in cellulo* [41, 42, 48, 49], although, by analogy with viroporins from alternative viruses, this may have diverse proviral functions [38]. For example, the 2B viroporin from coxsackievirus modulates calcium homeostasis, which leads to the suppression of apoptotic host cell responses [50]. Likewise, p7 may promote immune evasion by antagonizing the antiviral IFN function [51].

Interestingly, while p7 is released from the viral polyprotein through cleavage at E2-p7 and p7-NS2 junctions by the cellular signal peptidase [7, 52], it also exists in infected cells as an E2p7 precursor of poorly defined properties [7–11]. Intriguingly, virus mutants that exhibit either only E2p7 precursor (*i*.*e*., using a point mutation in E2-p7 cleavage site) or, conversely, no E2p7 expression (*i*.*e*., using an IRES sequence between E2 and p7) are both impaired for production of infectious particles [9, 14, 39, 53], suggesting that timely liberation of E2 and/or p7 are critical events for assembly/release of HCV particles.

Here, we explored how the retarded cleavage between E2 and p7 could regulate their functions associated to virion assembly and/or perturbation of cellular membrane processes. We demonstrate that p7 is able to regulate the cell secretory pathway, which induces intracellular retention of HCV glycoproteins, and to control release of nucleocapsid-free subviral and infectious viral particles. Specifically, through biochemical, imaging and functional analysis of a series of mutant viruses with modified E2-p7 junction as well as through p7 transcomplementation assays, our data uncover different mechanisms by which p7 regulates the proportion of different types of secreted HCV particles and determines their specific infectivity.

## Results

### HCV p7 inhibits the cell secretory pathway and induces E2 intracellular retention

**Fig 1:**
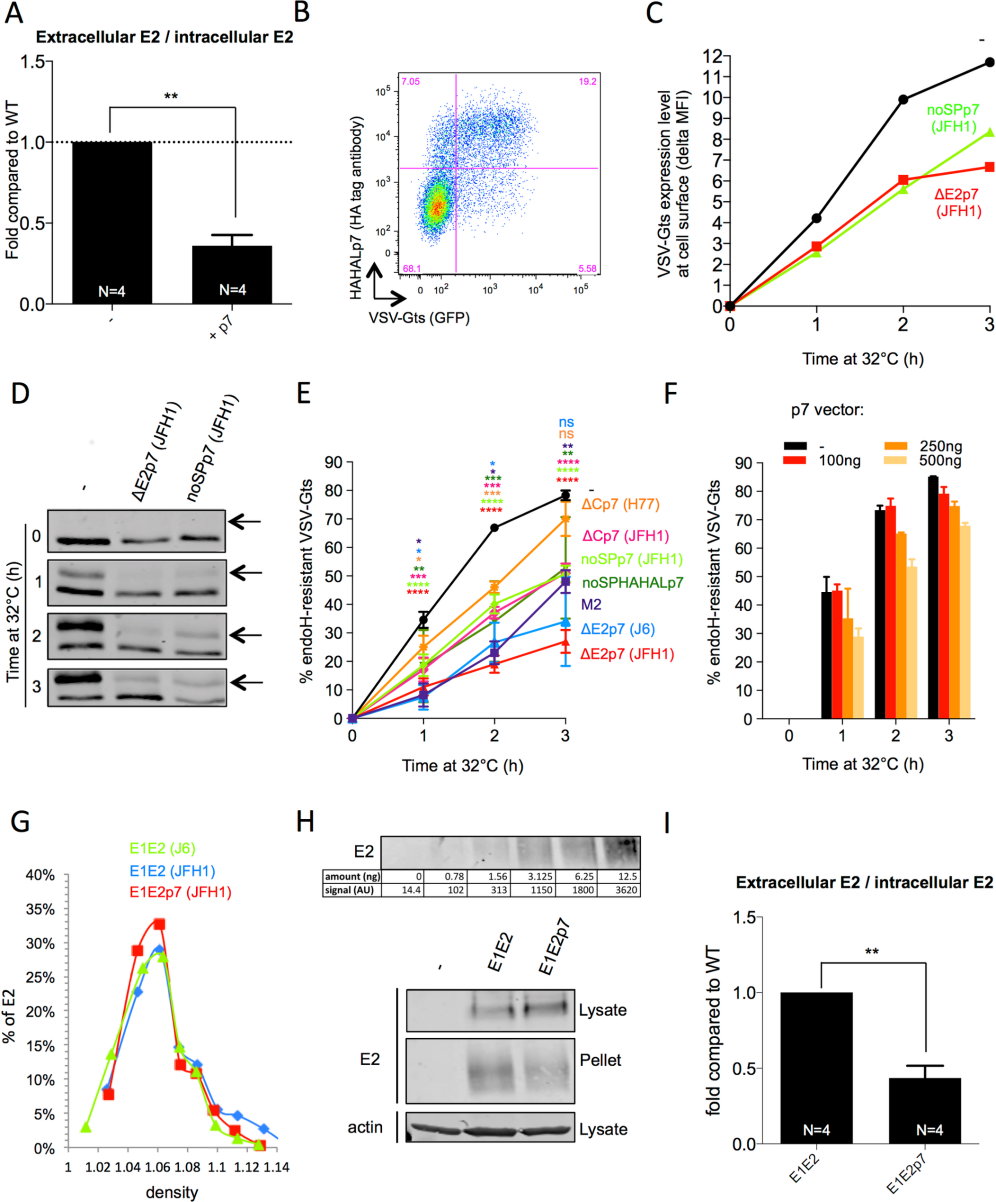
HCV p7 inhibits the cell secretory pathway. **(A)** Ratio of extra- *vs*. intra-cellular HCV E2 glycoprotein determined by western blot analysis of cell lysates and of pellets of ultracentrifuged supernatants from Huh7.5 cells co-electroporated with Jc1 HCVcc RNAs and p7-expression (+ p7) or control (-) vectors, at 72h post-electroporation. (**B**) Intracellular expression of VSV-Gts-GFP fusion protein (VSV-Gts) and HA-tagged p7 assessed by flow cytometry, at 24h post-transfection. **(C)** Cell surface expression of VSV-Gts assessed by flow cytometry, using the 41A1 mAb directed against VSV-G ectodomain, after co-transfection of Huh7.5 cells with vectors encoding VSV-Gts and p7 (JFH1) expressed with the end of E2 (∆E2p7) or no signal peptide (noSPp7). Transfected cells were grown overnight at 40°C, which maintains VSV-Gts unfolded and results in its accumulation in the ER. Cells were then incubated for different periods of time (0h, 1h, 2h and/or 3h, as indicated) at 32°C, which allows restoration of its folding and thus, its secretion. The values represent the variations of the mean fluorescence intensity (delta MFI) of cell surface-expressed VSV-Gts relative to time 0h at 32°C. **(D)** Representative western blot analysis of cell lysates co-expressing VSV-Gts and p7, digested with endoH glycosidase. Cells were grown overnight at 40°C and lysed at different time points after incubation at 32°C. The endoH-resistant VSV-Gts species (arrows) indicates proteins that traffic to and beyond the Golgi apparatus. **(E)** Quantification of western blot from independent experiments performed as described in (**D**) after co-transfection of VSVG-ts with different p7 or with influenza H7N1 M2 [97] protein-expressing constructs, as indicated. The values indicate the mean percentages of endoH-resistant VSV-Gts relative to total VSV-Gts for each time point. (**F**) p7 dose-dependence induction of endoH-resistance of VSV-Gts. **(G)** Representation of HCV E2 glycoprotein profile (expressed as percentage of total E2) in 3–40% iodixanol density gradients of subviral particles (SVP) produced after transduction of Huh7.5 cells with lentiviral vectors allowing the expression of either E1E2 or E1E2p7 proteins from J6 and/or JFH1 viruses. **(H)** Representative western blot of E2 detected with 3/11 antibodies in lysates and pellets of ultracentrifuged supernatants from cells producing SVPs (JFH1 strain). A range of dilutions of sE2 purified to homogeneity were run in parallel to the same western blot shown here. The intensity of E2 signals and the amounts of E2 are indicated. **(I)** Ratio of extra- *vs*. intra-cellular HCV E2 glycoprotein determined by quantification of western blots from independent experiments as described in (**H**). Data represent mean values ± SEM. The numbers of experiments performed are indicated below the graphs.

As HCV p7 is released through inefficient E2p7 cleavage, we first sought to address its function by increasing its expression levels in HCV-infected cells. We found that co-expression of individual p7 with JFH1 HCVcc RNAs in Huh7.5 hepatoma cells decreased the levels of extracellular particle- associated E2 proteins, resulting in *ca*. 3-fold reduced secretion (Fig 1A).

Since some viroporins from alternative viruses alter the canonical secretory pathway [38, 43], we then asked whether p7 could impact the secretion of VSV-G tsO45 (VSV-Gts), a temperature-dependent folding mutant of VSV-G glycoprotein commonly used as model cargo of protein secretion [54]. At 40°C, this protein remains unfolded, resulting in its accumulation in the ER, whereas its folding can be restored at 32°C, which allowed its transfer from the ER to the Golgi and then the plasma membrane (Fig 1C). We transfected in Huh-7.5 cells VSV-Gts with p7 constructs from different HCV strains and using different signal peptide configurations, which resulted in *ca*. 60% of cells co-expressing both proteins among the transfected cells (Fig 1B). As monitored by flow cytometry analysis, p7 co-expression significantly reduced the kinetics and levels of VSV-Gts cell surface expression at permissive temperature of 32°C (Fig 1C).

Next, to address how p7 alters traffic through the secretory pathway, we measured the resistance of intracellular VSV-Gts to endoH digestion, used as a marker of ER-to-Golgi traffic [54, 55]. While at 0h, all VSV-Gts glycans remained endoH-sensitive, reflecting ER retention at 40°C, they progressively became resistant to endoH cleavage upon 1-3h incubation at 32°C (Fig 1D–1F), underscoring VSV-Gts transfer to the Golgi apparatus. Importantly, p7 co-expression resulted in dose-dependent decrease of the kinetics of VSV-Gts endoH-resistance (Fig 1D–1F), in a manner similar to influenza virus M2 (Fig 1E), indicating that p7 slowed down the rate of VSV-Gts ER-to-Golgi traffic or, alternatively, favored its retention in the ER. We noticed that p7 proteins from different HCV genotypes/strains mediated this effect (Fig 1E) with that of H77 strain appearing less efficient for inhibiting Golgi transfer. We also found that p7 associated to its own signal-peptide, *i*.*e*., the E2 amino-terminus (∆E2p7 construct), induced the strongest VSV-Gts ER retention.

We then thought that p7-mediated alteration of the secretory pathway could induce the retention of HCV glycoproteins at ER membranes, which may favor assembly of HCV particles [21]. Thus, we expressed E1E2 glycoproteins, alone or with p7, to promote their secretion in the cell supernatant as subviral particles (SVP), *i*.*e*., nucleocapsid-free enveloped particles [24], that peaked at a buoyant density of *ca*. 1.05–1.06 g/ml (Fig 1G). As detected by E2 immunoblots from the pellets after ultracentrifugation of the cell supernatants (Fig 1H and 1I), we found that p7 co-expression induced up to 60% increase of E2 intracellular expression and concomitant 55% decrease of SVP release (Fig 1H). This resulted in a *ca*. 2–3 fold reduced E2 secretion (Fig 1I), which, combined with the results of Fig 1A, indicates that p7 can induce the retention of HCV glycoproteins.

### p7 N-terminal alterations that accelerate E2p7 cleavage inhibit HCV infectivity

**Fig 2:**
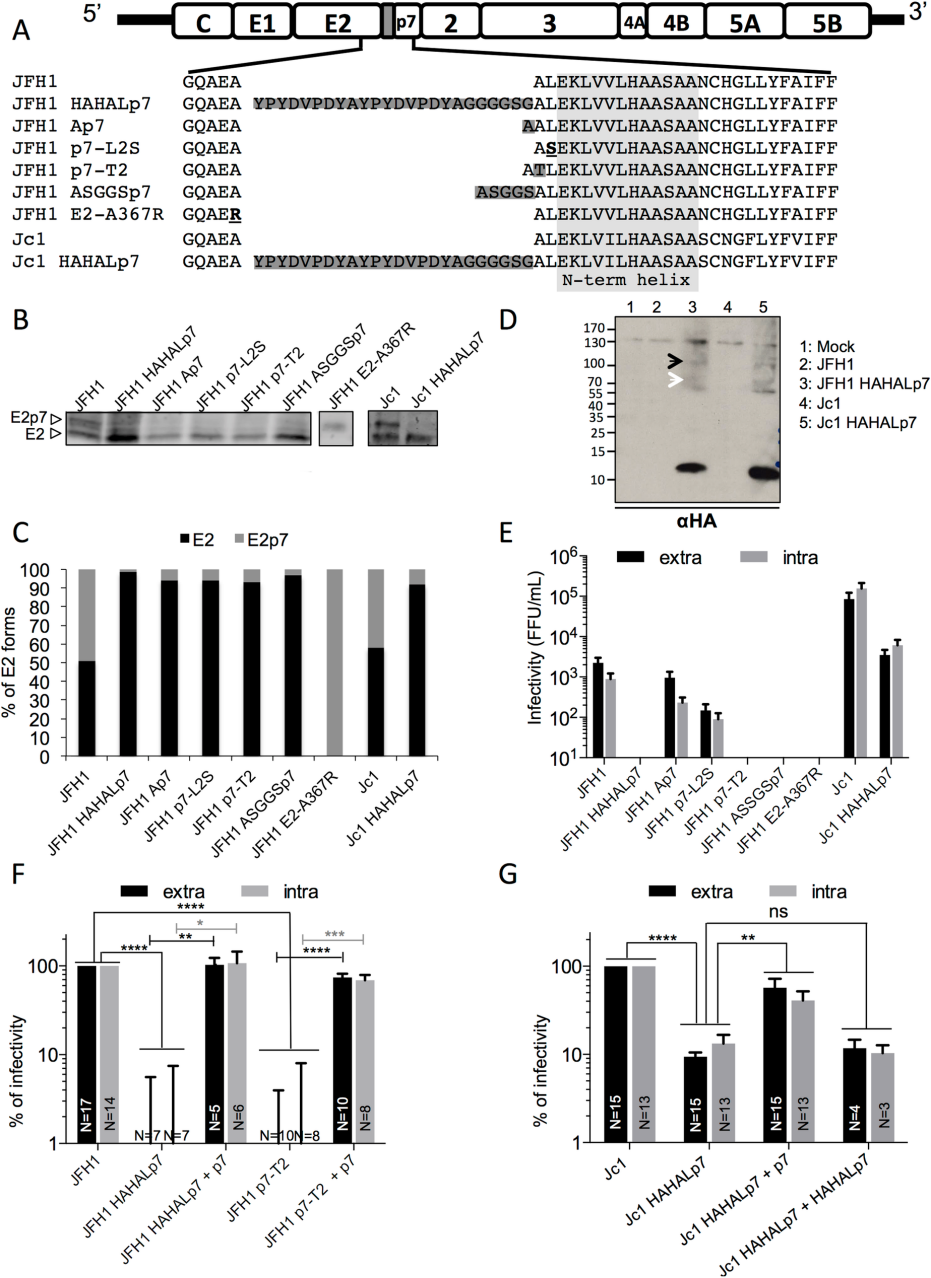
HCVcc mutants with modified p7 amino-termini prevent infectivity. **(A)** Schematic representation of JFH1 and Jc1 HCVcc mutants in E2-p7 junction. The first helix of p7 is shown in the light grey box. The insertions of residues are shown in dark grey and the substitutions are underlined. (**B-D**) Western blot analysis of Huh7.5 cells electroporated with RNAs from HCVcc viruses encoding wild-type or E2p7 cleavage mutants. **(B)** At 72h post-electroporation, cells were lysed and digested with endoH glycosidase before western blotting using 3/11 antibodies against E2. **(C)** The relative ratios of E2p7 precursors *vs*. free E2 species are indicated. **(D)** The HAHALp7 protein and the E2HAHALp7 (white arrows) and E2HAHALp7NS2 (black arrows) precursors were revealed using an HA tag antibody in lysates of cells electroporated with the indicated HCVcc constructs. **(E)** The extracellular (black) and intracellular (grey) infectivity levels for all mutants are represented. **(F)** The extracellular (black) and intracellular (grey) infectivity levels, normalized after determining the proportion of HCV-positive virus producer cells (see Fig 3A), of JFH1 HAHALp7 or p7-T2 mutant viruses expressed alone or with wild-type p7 (+ p7) are represented relative to infectivity of parental JFH1 virus. **(G)** The extracellular (black) and intracellular (grey) infectivity levels, normalized by determining the proportion of HCV-positive virus producer cells, of Jc1 HAHALp7 mutant virus expressed alone or with wild-type p7 or HAHALp7, as indicated, are represented relative to infectivity of parental Jc1 virus. Data are displayed as means ± SEM. The numbers of experiments performed are indicated below the graphs.

By controlling the release of free p7, E2p7 cleavage efficiency may adjust the levels of p7 expression, which could regulate the extent by which p7 slows down the cell secretory pathway and, concomitantly, the traffic and thus secretion of HCV E2 glycoproteins (Fig 1A, 1H and 1I). Hence, we introduced mutations at the E2-p7 junction in Jc1 and/or JFH1 viruses in order to design HCVcc mutants that exhibit increased E2p7 cleavage scores (Fig 2A), as predicted by SignalP method (http://www.cbs.dtu.dk/services/SignalP/). This strategy was preferred over the use of an IRES sequence between E2 and p7, as reported before [9, 10, 14], because it induces a natural, *i*.*e*., signal peptidase-mediated liberation of p7 from the HCV polyprotein. First, we inserted linkers of various sizes at the N-terminus of p7 (HAHALp7 and ASGGSp7 viruses), which left p7 sequence unchanged; the former linker consisting of a double HA tag allowing the detection of p7 [56]. Second, we introduced a substitution at position 2 of p7 (p7-L2S). Third, we generated p7 mutants having insertions of a single residue, either a threonine (p7-T2) at position 2 [8] or an alanine (Ap7) at position 1 of p7 (Fig 2A). None of these mutations–termed hereafter p7 ATMI (Amino-Terminus Membrane Interface) mutants, introduced before the first p7 helix (shown as grey box in Fig 2A) [30, 35, 36], are expected to change p7 structure or opening of its channel pore, as shown by molecular modeling (S1 Fig). Of note, we could not identify mutations at E2 carboxy-terminus that accelerated E2p7 cleavage. Finally, we also introduced a control mutation abrogating E2p7 cleavage (E2-A367R; Fig 2A) [9].

Next, we investigated the rate of E2p7 cleavage by treating lysates of Huh-7.5 cells expressing mutant virus RNAs with EndoH to remove E2 glycans, which improved E2 *vs*. E2p7 electrophoretic separation (Fig 2B and 2C). Except for the E2-A367R mutant that was not cleaved, as expected, all mutants displayed almost complete E2p7 cleavage, which compared to the *ca*. 40% and 50% uncleaved E2p7 precursor detected with parental Jc1 and JFH1 viruses, respectively. Using a HA-tag antibody to reveal the HAHALp7 protein, we confirmed that the accelerated cleavage detected for the JFH1 HAHALp7 and Jc1 HAHALp7 viruses induced the release of p7 at the expected molecular size with poor if not undetectable E2p7 (*i*.*e*., E2HAHALp7) expression (Fig 2D), though such analysis could not be extended to the other p7 ATMI mutants owing to the unavailability of antibodies against native p7. In addition, as previously reported [8, 11], we also detected small amounts of E2p7NS2 precursor for both wt and mutant viruses (Fig 2D; S2A Fig).

Interestingly, when we investigated the infectivity of these mutant viruses, we found that, relative to parental viruses, the p7 ATMI mutant viruses had decreased extracellular infectivity, by *ca*. 3-fold to over 100-fold (Fig 2E), depending on the p7 modifications and the virus backbones (Fig 2A). Particularly, the Ap7 insertion and the p7-L2S substitution mutants in JFH1 virus induced *ca*. 3- and 10-fold decreased infectivity, respectively, whereas the p7-T2, ASGGSp7, and HAHALp7 JFH1 insertion mutants exhibited complete loss of infectivity (Fig 2E). Likewise, the Jc1 HAHALp7 mutant virus displayed a 10–20 fold reduced infectivity, as compared to parental virus (Fig 2E). Similar defects were observed for intracellular infectivity (Fig 2E), indicating that these p7 ATMI mutant viruses were not impaired at the stage of secretion of viral particles. Finally, we found that co-expression of wt p7 (though not mutant p7 such as HAHALp7) restored the production of both extracellular and intracellular infectious particles to levels detected for wt viruses (Fig 2F and 2G; S2B Fig). Hence, since all p7 ATMI mutants displayed nearly complete E2p7 cleavage (Fig 2B and 2C) and since HAHALp7 co-expressed with mutant viruses did not restore infectivity (Fig 2G), these results indicated that the integrity of the N-terminal end of p7 itself is crucial for assembly of infectious particles.

Thus, in the subsequent experiments, we compared more particularly the Jc1 HAHALp7 virus to its parental Jc1 counterpart since this mutant retained some infectivity levels, which allowed us to further characterize this novel phenotype; yet, the most salient results described below could be extended to the other p7 ATMI mutant viruses (see supplemental figure set).

### p7 modulates the expression and secretion of HCV particle components

**Fig 3:**
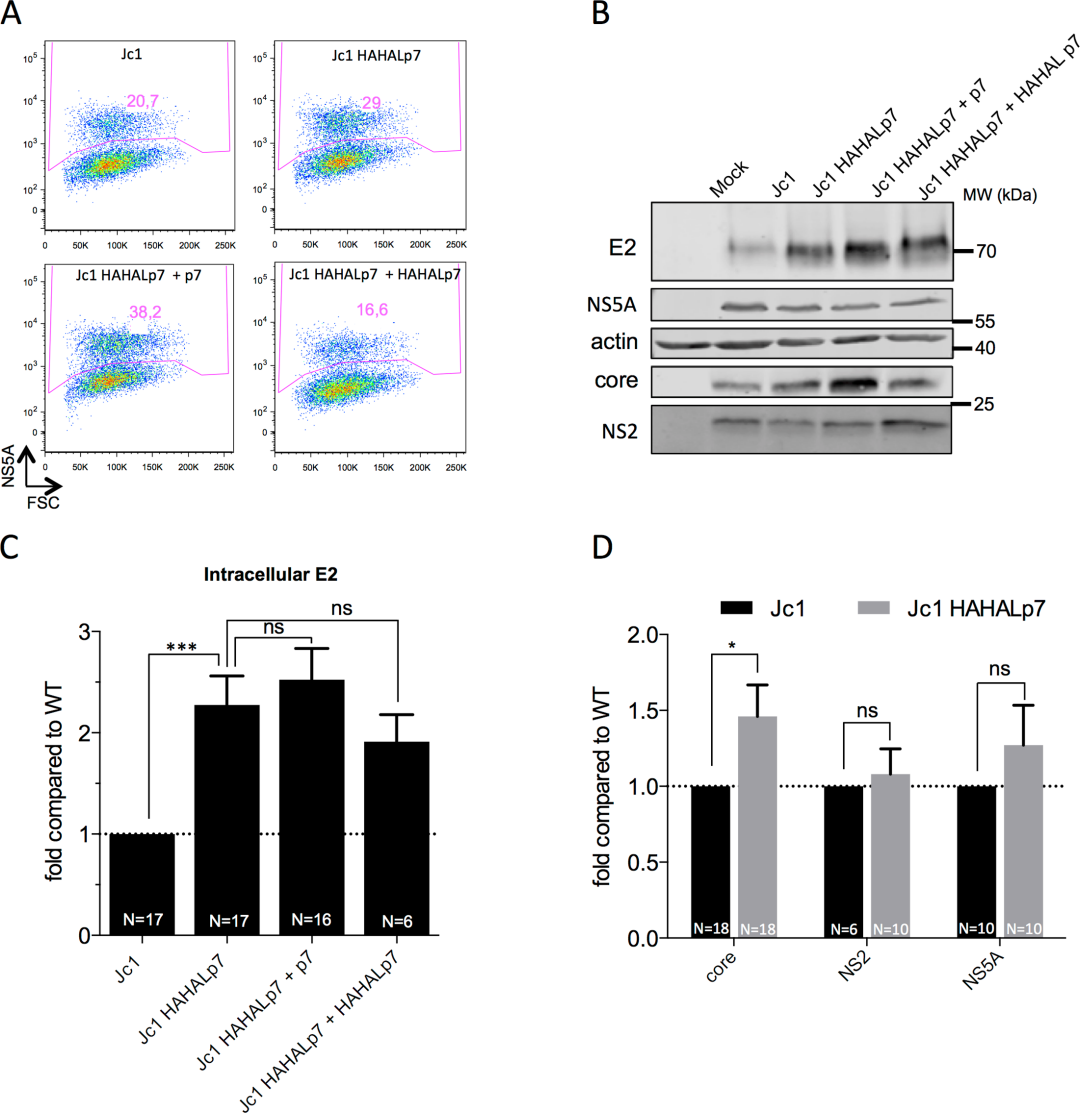
p7 ATMI mutant viruses increase the expression levels of HCV structural proteins. Huh7.5 cells were electroporated with RNAs from parental or Jc1 HAHALp7 mutant viruses expressed alone or with wild-type p7 or HAHALp7. Analyses were performed at 72h post-electroporation. Results obtained with other p7 ATMI mutant viruses are shown in S3 Fig. **(A)** Flow cytometry analysis of HCVcc-expressing cells with NS5A antibody used to determine the proportion of HCV-positive virus producer cells. **(B)** Representative western blot analysis of cell lysates using antibodies against the indicated proteins. **(C)** Quantification of intracellular E2 levels. **(D)** Quantification of intracellular levels of core, NS2 and NS5A proteins. **(C-D)** Proteins were quantified and normalized after determining the proportion of HCV-positive virus producer cells (as determined in (**A**)) and the amounts of cellular actin. The values are displayed relative to expression of E2, core, NS2 and NS5A in Jc1 HCVcc RNA-electroporated cells. Data represent mean values ± SEM. The numbers of experiments performed are indicated below the graphs.

Since p7 ATMI mutant viruses displayed augmented E2p7 cleavage rates (Fig 2), we wondered whether they had altered E2 expression levels. As compared to wt viruses, we observed a 2–3 fold increased intracellular expression of total E2 (*i.e.*, free E2 + E2p7) for all mutant viruses exhibiting increased E2p7 cleavage (Fig 3A–3C; S3A Fig). No modification of E2 half-life could be detected for mutant *vs*. wt virus (S3C Fig). Specifically, taking into account that *ca*. 40% E2 was detected as E2p7 precursor for wt JC1 virus (Fig 2B and 2C), we estimated that the actual ratio of free E2 expression for mutant *vs*. wt viruses is *ca*. 4–5 fold. We also found that core expression was increased by *ca*. 1.5–2 fold (Fig 3B and 3D; S3B Fig), whereas expression levels of NS2 and NS5A non-structural proteins (Fig 3B and 3D) were unchanged. As similar results were obtained for all p7 ATMI mutant viruses, this indicated that these mutations modulated the expression levels of structural proteins without altering viral replication and/or translation. Importantly, co-expression of either wt p7 or HAHALp7 did not revert E2 expression to wt levels (Fig 3B and 3C), which indicated that increased E2p7 cleavage (rather that p7 N-terminus modification *per se*) induce up-regulation of E2 glycoproteins.

**Fig 4:**
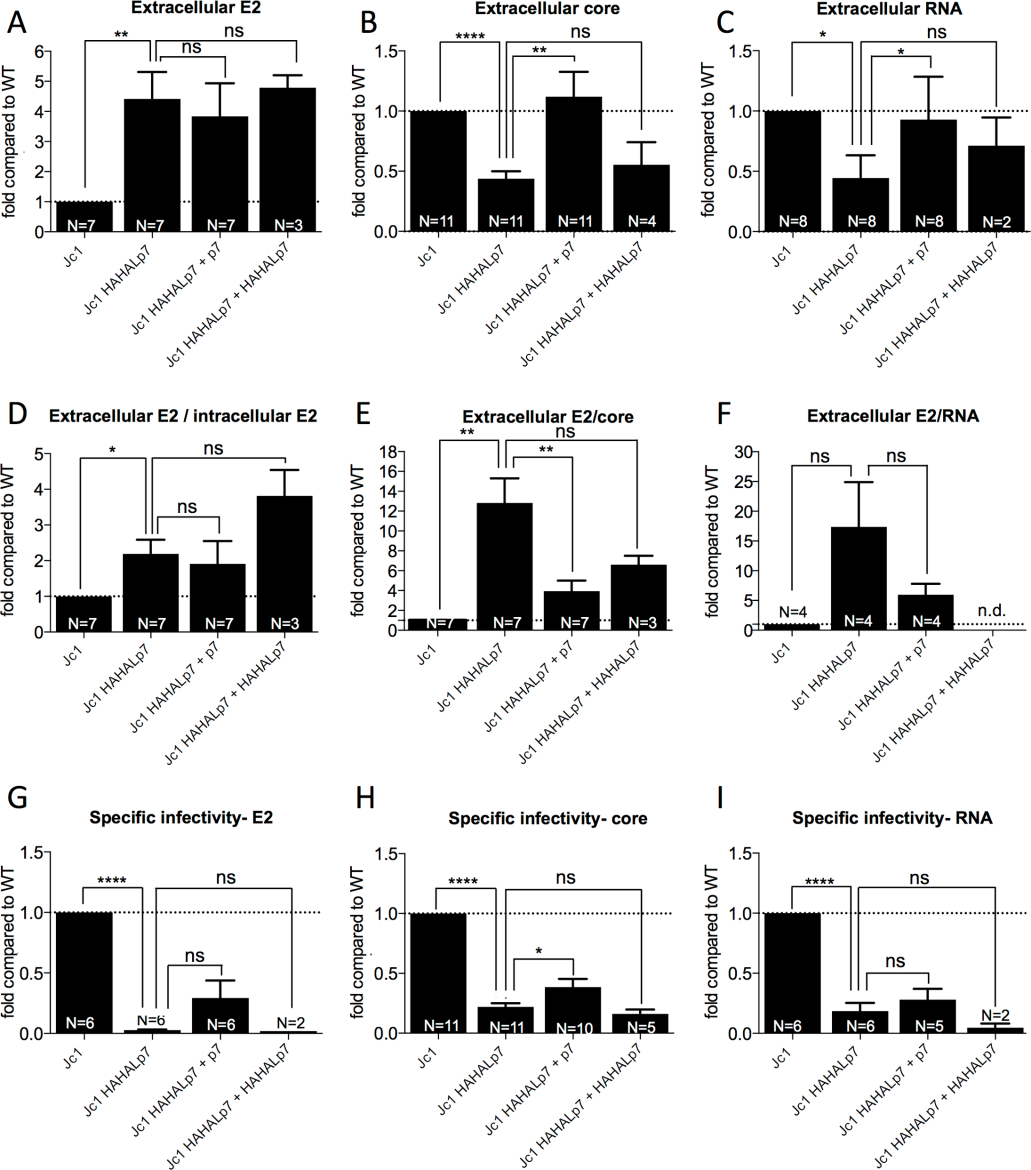
p7 ATMI mutant viruses display increased E2 secretion and decreased RNA and core secretion. Huh7.5 cells were electroporated with RNAs from parental or Jc1 HAHALp7 mutant HCVcc viruses expressed alone or with wild-type p7 or HAHALp7. Analyses were performed at 72h post-electroporation. All data are normalized by percentage of HCV-positive virus producer cells obtained as in Fig 3A. Results obtained with other p7 ATMI mutant viruses are shown in S4 Fig and S5 Fig. (**A**) Levels of secreted E2 determined by quantitative western blot following GNA lectin pull down of cell supernatants. (**B**) Levels of secreted core as determined by CMIA (Chemiluminescent Microparticle ImmunoAssay). (**C**) Levels of secreted HCV RNAs as determined by RT-qPCR. (**D**) Ratio of extracellular E2 to intracellular E2. (**E**) Ratio of extracellular E2 to extracellular core. (**F**) Ratio of extracellular E2 to extracellular HCV RNA. (**G**) Specific infectivity relative to E2 amounts. (**H**) Specific infectivity relative to core amounts. (**I**) Specific infectivity relative to RNA amounts. All values are displayed relative to infectivity and expression of E2, core or RNA values determined in the supernatants of Jc1 virus-electroporated cells (**A-I**). Data represent mean values ± SEM. The numbers of experiments performed are indicated below the graphs.

Next, we hypothesized that the increased expression of structural proteins could be due to a blockage of their secretion, which may also explain the losses of mutant virus infectivity (Fig 2E–2G). Thus, we quantified the total secretion of virion components, *i*.*e*., E2 glycoproteins, core and viral RNAs, in the supernatants of cells expressing p7 ATMI mutant virus relative to wt virus (Fig 4). Using immuno-precipitation (IP) assays of cell supernatants with GNA lectins, which bind glycans present on HCV E1E2 glycoproteins [57], we detected a *ca*. 4–5 fold increased secretion of E2 protein (Fig 4A), which matched the 4–5 fold elevated levels of intracellular free E2 (Fig 3C, combined with Fig 2B and 2C). Although the ratio of extracellular *vs*. total intracellular E2 expression levels indicated a 2–3 fold difference between wild-type and mutant viruses (Fig 4D), taking into account that 40% of intracellular E2 species were in the form of non-secreted E2p7, we deduced identical ratios of extracellular *vs*. intracellular free E2 for wt and mutant viruses. Similar results were obtained for the other p7 ATMI mutants (S4A Fig), suggesting that the increased E2 secretion from cells expressing the p7 ATMI mutant viruses is directly linked to the augmented intracellular E2 expression. As co-expression of p7 in trans did not significantly restore E2 expression (Fig 3C) and secretion (Fig 4A and 4D) to wt levels, this indicated that the delayed cleavage of E2p7 is essential for the control of intracellular and extracellular E2 levels.

Strikingly, in contrast to E2, we observed that the p7 ATMI mutant viruses had decreased secretion of both core and viral RNAs in the cell supernatants, by *ca*. 2–5 fold (Fig 4B and 4C; S4B and S4C Fig). Furthermore, we found that wt p7 restored normal secretion levels of nucleocapsid components (Fig 4B and 4C; S4D Fig) though not those of E2 glycoproteins (Fig 4A). This indicated that viruses harboring p7 ATMI mutations display differential alterations of pathways leading to trafficking and/or secretion of viral glycoproteins *vs*. nucleocapsid components, *i*.*e*., HCV core and RNA. Finally, since mutant p7, *e*.*g*., HAHALp7, co-expression did not restore normal secretion levels of core and RNA (Fig 4B and 4C), these results indicated that p7 itself, rather than its cleavage from E2, modulates the secretion of viral nucleocapsids.

Altogether, our results suggest that altered p7 expression, as induced by accelerated cleavage and release from E2 as well as by its N-terminal modification, influence the proportion of secreted virion components. Indeed, relative to core and/or viral RNAs, a 15–25 fold higher expression of HCV glycoproteins was detected in the supernatants of cells infected with Jc1 HAHALp7 virus as compared to wt virus (Fig 4E and 4F).

Interestingly, we found that the p7 ATMI mutant viruses exhibited strongly decreased specific infectivity relative to their content in core protein or viral RNA, from 4–5 fold for Jc1 HAHALp7 virus (Fig 4H and 4I) to over 50-fold for other mutants (S5A and S5B Fig). Likewise, relative to E2 glycoprotein, the specific infectivity of the Jc1 HAHALp7 virus was decreased by *ca*. 35-fold, as compared to the parental virus (Fig 4G). Importantly, co-expression of wt p7, but not of ATMI mutant p7, restored (though not completely), the specific infectivity of the mutant viruses (Fig 4G–4I; S5C Fig). Altogether, this pointed out to altered ratios of different secreted forms of HCV-derived particles incorporating these components or, alternatively, altered composition of the viral particles themselves.

### p7 controls secretion levels of HCV-derived particles

**Fig 5:**
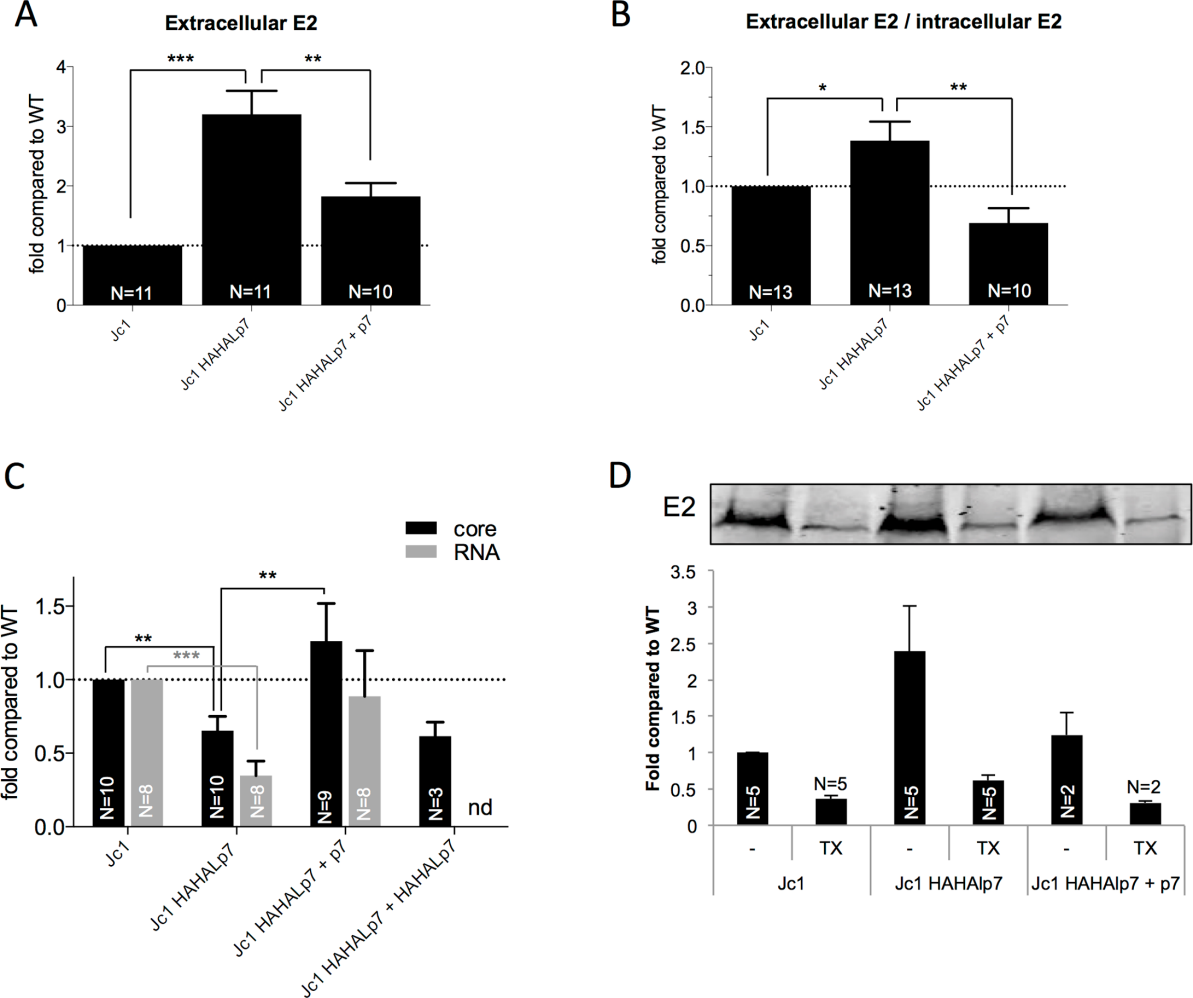
p7 ATMI mutant viruses modulate levels of particle-associated viral components. Huh7.5 cells were electroporated with RNAs from parental *vs*. Jc1 HAHALp7 mutant viruses expressed alone or with wild-type p7 or HAHALp7, as indicated. Analyses were performed at 72h post-electroporation and normalized by percentage of HCV-positive virus producer cells obtained as in Fig 3A. Results obtained with other p7 ATMI mutant viruses are shown in S6 Fig. (**A**) Levels of E2 in pellets from ultracentrifuged cell supernatants. (**B**) Ratio of E2 in pellets *vs*. intracellular E2. (**C)** Levels of core and HCV RNA present in the pellets of ultracentrifuged cell supernatants as determined by CMIA and RT-qPCR, respectively. **(D)** Cell supernatants were incubated with 1% Triton X-100 (TX) or left untreated (-) before ultracentrifugation and analysis of E2 present in the pellets analyzed by quantitative western blot. All values are displayed relative to E2, core or RNA values determined in the pellet of supernatants from Jc1 virus-electroporated cells (**A-D**). Data represent mean values ± SEM. The numbers of experiments performed are indicated below the graphs.

To demonstrate if the viral components were secreted in particulate forms, we centrifuged the supernatants of infected-cells in conditions allowing sedimentation of particles. We detected in the pellets a *ca*. 3-fold increase of E2 levels (Fig 5A; S6A Fig) and a 2–3 fold decrease of both core and RNA (Fig 5C) while comparing Jc1 HAHALp7 mutant *vs*. parental viruses. This augmentation of secreted particle-associated E2 levels could be detected for all p7 ATMI mutant viruses (S6B Fig). Interestingly, we also observed an increased secretion of E1-containing particles (S6A Fig), likely owing to secretion of HCV glycoproteins as E1E2 heterodimers. Furthermore, ectopic expression of wt p7 restored wt levels of particle-associated HCV glycoproteins, core and RNA (Fig 5A and 5C). Since ectopically-expressed ATMI mutant p7 did not rescue the above levels (Fig 5C), this indicated that p7 N-terminus modifications rather than accelerated E2p7 cleavage induced changes in secretion levels of mutant viral particles, perhaps by altering the ratios between SVPs and (infectious) viral particles.

Then, we aimed at characterizing the different types and proportions of secreted particles for wt *vs*. p7 ATMI mutant viruses.

First, to confirm that E2 detected in the pellets of ultracentrifuged cell supernatants was secreted as particles, we treated the supernatants of virus-expressing cells with Triton-X100 before ultracentrifugation. We found that such treatment decreased the presence of E2 in the pellets for both wt and mutant viruses (Fig 5D; S6C Fig), hence indicating that a substantial part of secreted E2 proteins were in a sediment form, likely vesicular.

**Fig 6:**
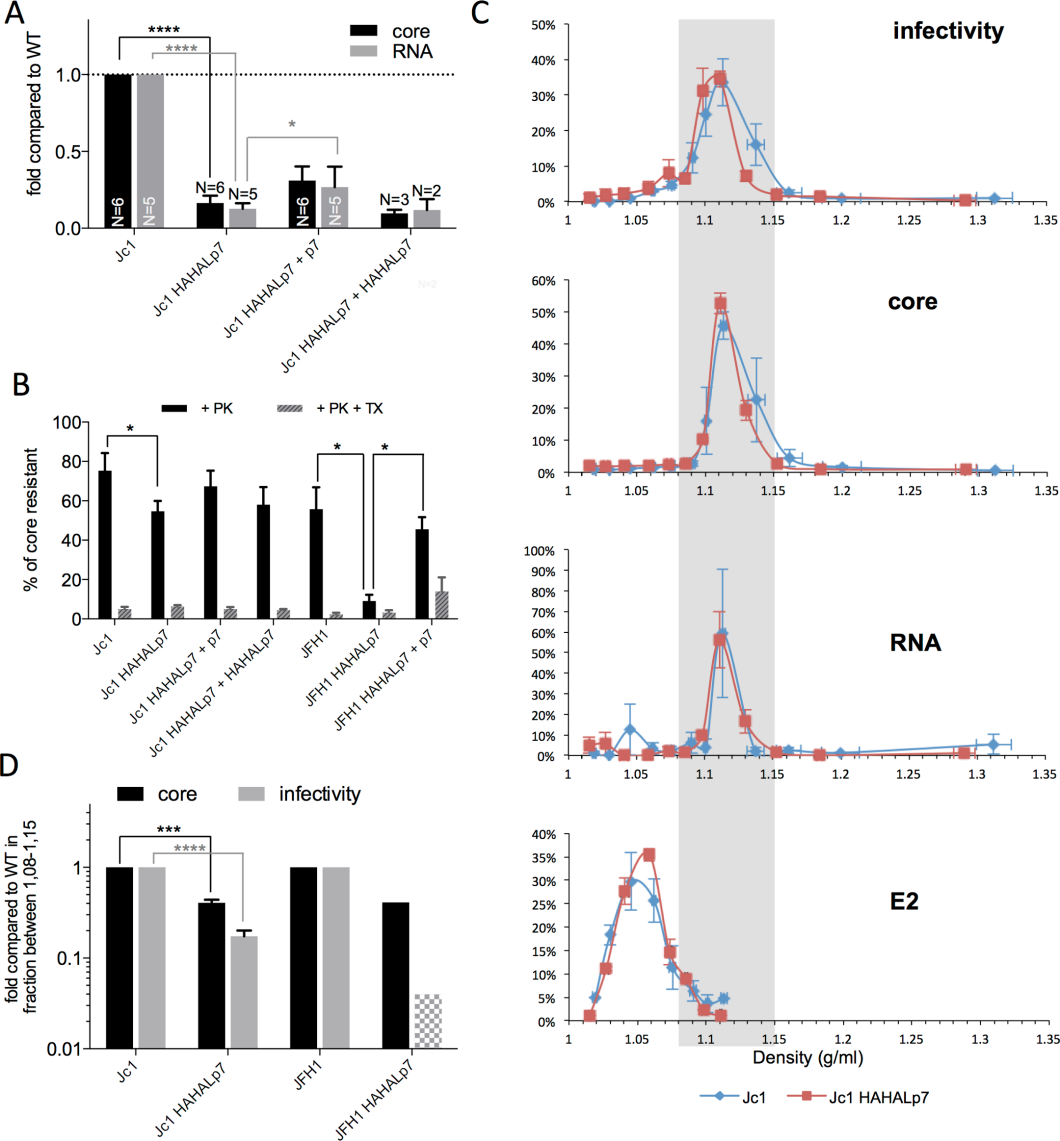
p7 ATMI mutant viruses induce secretion of viral particles with impaired envelopment. Huh7.5 cells were electroporated with RNAs from parental *vs*. Jc1 HAHALp7 or JFH1 HAHALp7 mutant viruses expressed alone or with wild-type p7 or HAHALp7, as indicated. Analyses were performed at 72h post-electroporation. **(A)** Levels of core and RNA following GNA pull down of cell supernatants, as determined by CMIA and RT-qPCR, respectively, and normalized by the amounts of immuno-precipitated E2. The values are displayed relative to association of core or RNAs to E2 pulled-down from the supernatants of Jc1 virus-electroporated cells. **(B)** Cell supernatants were digested with proteinase K (+ PK) with (+ TX) or without pre-treatment with Triton X-100 and the residual amounts of core (*i*.*e*., lipid membrane-protected core) were determined by CMIA. The values are displayed relative to non-treated conditions. **(C)** Analysis of density gradients of cell supernatants. Infectivity, core, RNA, and E2 were measured in each fraction and expressed as percentage of the sum of fractions. Examples of raw data of infectivity can be found in S7A–S7C Fig. **(D)** Infectivity and core of the indicated viruses were measured in pooled fractions of densities of 1.08–1.15 g/ml and represented as data normalized by values obtained with parental viruses. The grey shaded area represents values below the sensitivity threshold of the experiments. Data represent mean values ± SEM.

Second, since the p7 ATMI mutant viruses secreted higher E2 amounts with poorer infectivity compared to wt viruses (Fig 4G), we quantified the association of their glycoproteins with other virion components, *i*.*e*., core and RNA. When we pulled down E2 using GNA lectins, we found a 5–6 fold decreased association of core and RNA with E2 (Fig 6A). Moreover, ectopic expression of wt p7, though not mutant p7, restored, though partially, the association of E2 with viral core and genome (Fig 6A).

Finally, we investigated the degree of envelopment of the secreted core proteins within a lipid bilayer. Hence, we treated the supernatants of virus-expressing cells with proteinase K and subsequently determined the amounts of proteinase K-resistant core, which indicates its full protection by a lipid membrane or, conversely, its secretion as naked or badly enveloped core particles. Interestingly, as compared to their corresponding parental viruses, we detected decreased amounts of membrane-protected core for Jc1 HAHALp7 virus and, more dramatically, for JFH1 HAHALp7 virus (Fig 6B), which correlated with their respective losses of infectivity (Fig 2E–2G). Furthermore, we found that co-expression of wt p7, though not mutant p7, restored the lipid membrane envelopment of their secreted core proteins to almost wt levels (Fig 6B).

Altogether, these results pointed out to a disruption induced by p7 amino-terminus changes of the degree of association between HCV glycoproteins, viral envelopes and nucleocapsids, which could be due to an excess of E2 *vs*. core and RNA forms secreted independently, such as SVPs *vs*. naked/partially enveloped core particles, respectively, or, alternatively, to an increased density of E2 glycoproteins on the surface of secreted viral particles.

To investigate this further, we separated virus sub-populations using buoyant density-gradient fractionation. Jc1 and JFH1 HCVcc physical particles had more than 90% of viral RNA and 95% of core protein in fractions of densities of 1.10–1.15 g/ml, with a peak at 1.11 g/ml (Fig 6C; S7A–S7C Fig). As shown before [58–62], core and RNA could also be detected at higher densities (to up to 1.36 g/ml) and at lower densities, until 1.02 g/ml (S7 Fig). Jc1 HCVcc particles had more than 80% of their infectivity in fractions of densities of 1.08–1.13 g/ml, with a peak at 1.11 g/ml (Fig 6C; S7A and S7B Fig), in agreement with recent reports [57, 60–64]. Lastly, we found that E2 glycoproteins were detected in lower density fractions, with *ca*. 90% in fractions of densities of 1.03–1.08 g/ml and a peak at 1.05–1.06 g/ml (Fig 6C; S7A Fig) representing SVPs (Fig 1G). Importantly, less than 10% of E2 could be detected at densities of 1.08–1.15 g/ml, in which physical and infectious particles were prominent and corresponded to enveloped viral particles.

Interestingly, the density profile of Jc1 HAHALp7 virus was qualitatively similar to that of wt virus (Fig 6C; S7A and S7B Fig) and did not reveal any alteration of the distribution of the different types of particles along the gradient. However, quantitatively, we found the same alterations of the ratios of E2, core, RNA and infectivity in the different fractions for the mutant *vs*. parental virus (S7A Fig), as compared to unfractionated viral particles (Fig 5A and 5C). Specifically, 2–3 fold augmented E2 levels were detected in the SVP fractions of 1.03–1.08 densities (S7A Fig). Likewise, 2–3 fold reduced core or RNA levels were detected in all fractions whereas infectious titers were decreased by *ca*. 10-fold in these fractions (S7A Fig). Furthermore, we found that, whatever the density, the co-expression of Jc1 HAHALp7 virus with ectopic p7 restored wt infectivity concomitantly to restoration of the wt levels of core and RNA (S7B Fig).

Finally, we found that the loss of infectivity was accentuated for the JFH1 HAHALp7 virus, which displays a stronger phenotype than the Jc1 HAHALp7 virus (Fig 2E–2G), in fractions of densities of 1.08–1.15 g/ml containing most viral particles (Fig 6C; S7C Fig). Particularly, while core levels were reduced by *ca*. 2–3 fold for both Jc1 HAHALp7 and JFH1 HAHALp7, 6- and over 100-fold reductions of infectivity levels were detected for the Jc1 HAHALp7 and JFH1 HAHALp7 mutant viruses, respectively, relative to parental viruses (Fig 6D).

Altogether, these results suggested that the p7 N-terminus controls both the secretion and the specific infectivity of secreted virus particles, likely *via* a process involving the completion of their envelopment, as indicated by the PK-sensitivity of core from secreted p7 ATMI mutant particles.

### p7 amino-terminal alterations impair NS2, NS5A and E2 interactions required for envelopment of infectious particles

We then aimed at dissecting how p7 modulates the composition of viral particles by addressing HCV assembly mechanisms, from intracellular clustering of virion components to their envelopment.

First, since the HCV virion assembly rate is linked to core subcellular localization [17, 65–67] and since p7 alters this event in concert with NS2 [17, 40], we investigated by confocal microscopy analysis whether core from p7 ATMI mutant viruses could be relocated from lipid droplets to ER membranes, where envelopment and release of viral particles occur [3, 21]. While individually expressed Jc1 core had predominant distribution around lipid droplets, as previously described [17], its co-expression with either wt p7 or HAHALp7 protein induced full targeting at ER membranes (S8A Fig). Similar results were obtained with full-length viruses, since no difference between Jc1 and Jc1 HAHALp7 viruses could be detected regarding the prevalent ER-localization of their core proteins (S8B Fig). These data indicated that the p7 ATMI mutations and/or the accelerated E2p7 cleavage did not prevent p7-mediated early assembly events leading to core targeting to the ER membrane, but rather, impaired later assembly events.

**Fig 7:**
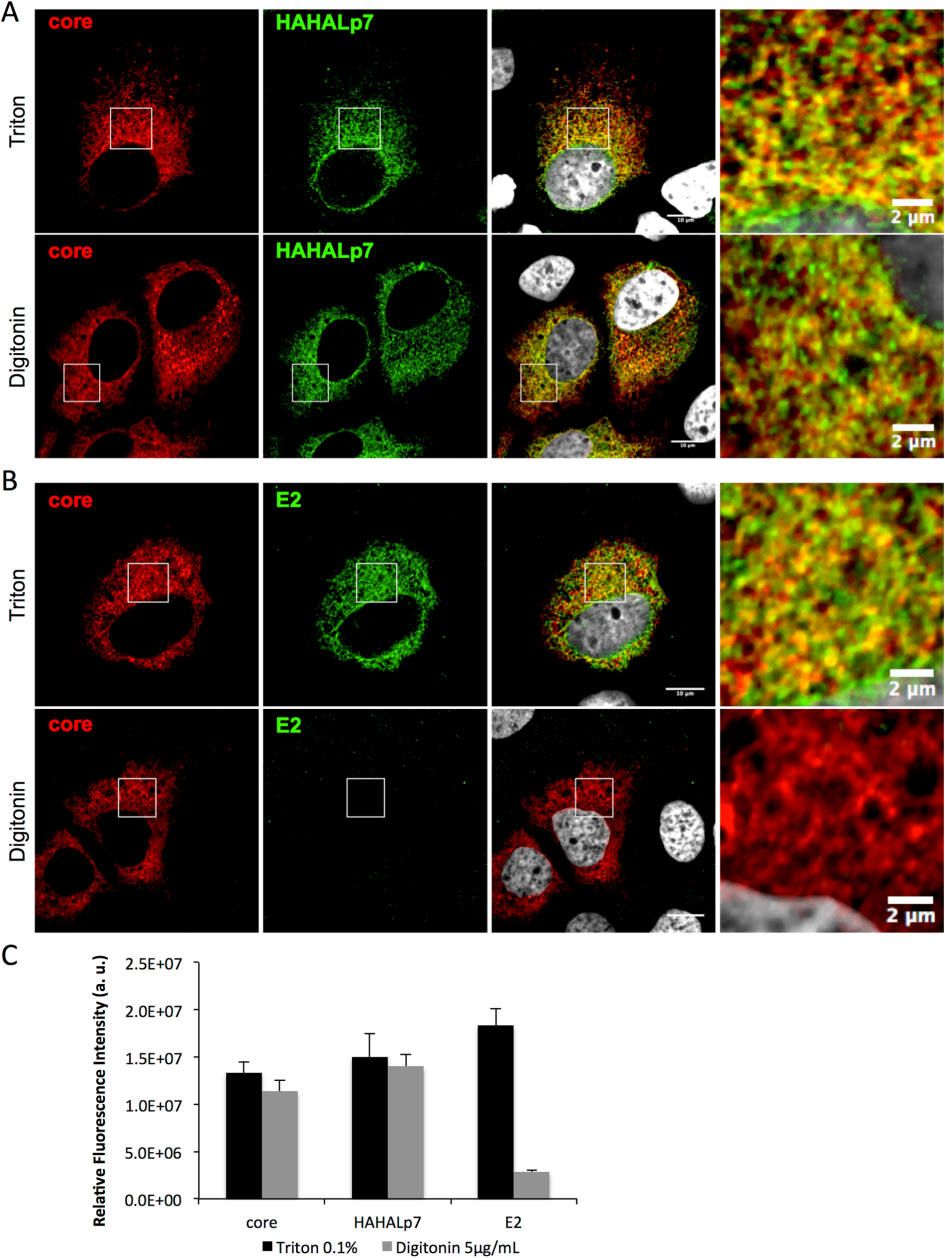
Co-localization of p7 ATMI mutant and E2 with core protein in HCVcc infected cells. Confocal microscopy analysis of Huh7.5 cells infected with Jc1 HAHALp7 virus. At 72h post-infection, cells were fixed, permeabilized with either Triton X-100 or Digitonin, as indicated, and stained for HCV core (red), HAHALp7 (green) (**A**), E2 (green) (**B**) and nuclei (grey). The relative fluorescence intensity (arbitrary units) of each channel (**C**) was quantified by using ImageJ.

Using HA-tag antibodies, we confirmed that HAHALp7 and core co-localized at the ER in cells infected with the Jc1 HAHALp7 virus (Fig 7A), as reported before [56]. Interestingly, we found that HAHALp7 and core co-localization could also be detected in infected cells treated with 5μg/ml digitonin (Fig 7A and 7C), which permeabilizes plasma but not ER membranes [68, 69]. Similar results were also obtained with JFH1 HAHALp7 virus as well as with HAHALp7 expressed individually, with or without signal peptide (S9A–S9C Fig). Since E2/core co-localization could be detected in Triton-treated cells but not in digitonin-treated cells (Fig 7B and 7C; S9A–S9C Fig), as expected owing to the luminal exposition of E2 ectodomain, these results indicated that the N-terminus of HAHALp7 points towards the cytosol. Note that these results do not exclude that p7 may also adopt the reverse topology, *i*.*e*., with N- and C-termini exposed toward the luminal side of the ER [70].

**Fig 8:**
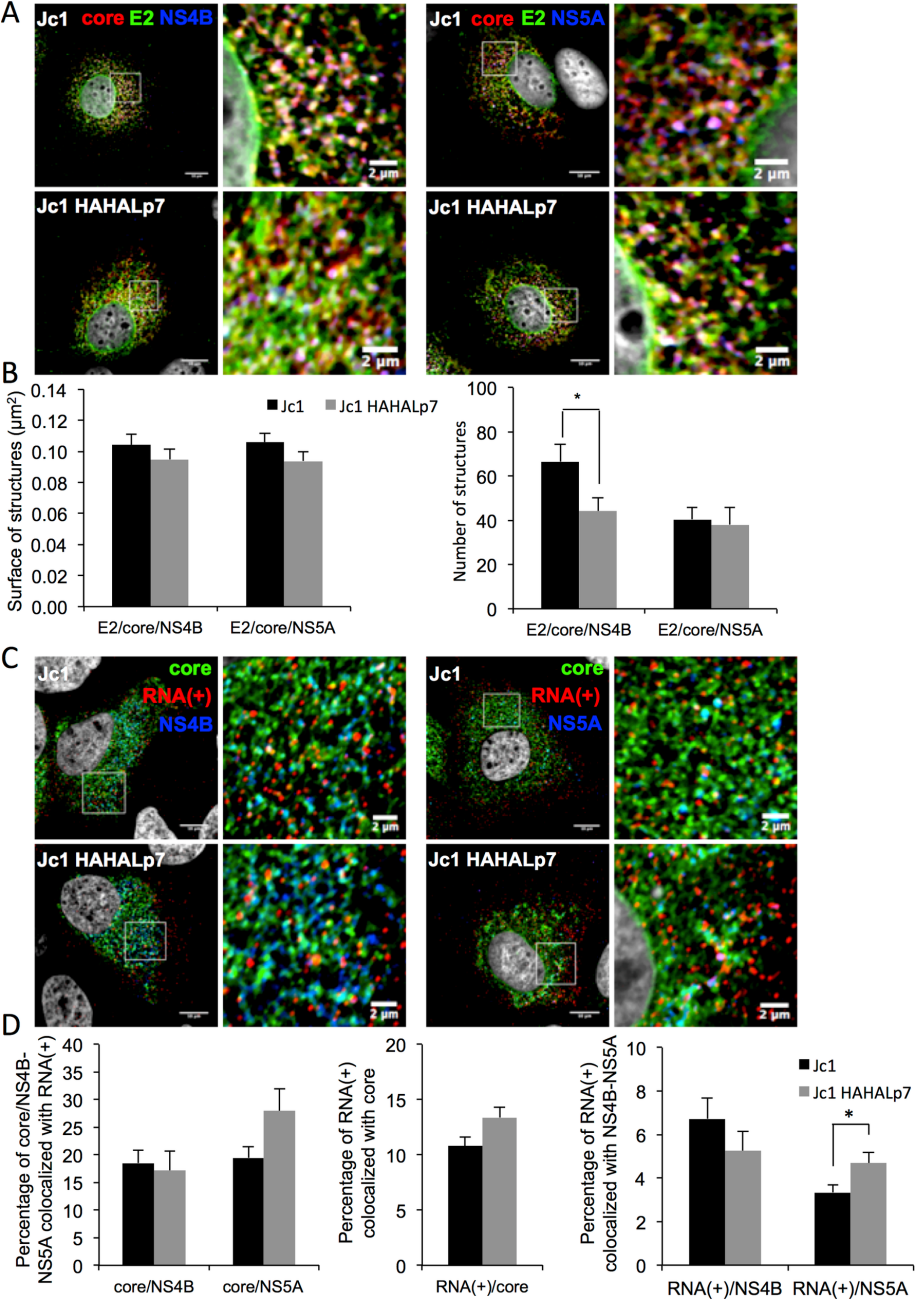
p7 ATMI mutant viruses do not alter E2, core, NS4B, NS5A and HCV RNA(+) clustering. **(A)** Confocal microscopy of Huh7.5 cells infected with Jc1 or Jc1 HAHALp7 viruses. At 72h post-infection, cells were fixed and stained for HCV core (red), E2 (green), NS4B (blue, left panels), NS5A (blue, right panels) and nuclei (grey). **(B)** Quantification of size (left) and number (right) of co-localized core/E2/NS4B or core/E2/NS5A structures. (**C**) Confocal microscopy of Huh7.5 expressing Jc1 or Jc1 HAHALp7 viruses. At 72h post-infection, cells were fixed and stained for HCV core (green), NS4B (blue, left panels), NS5A (blue, right panels) and nuclei (grey). HCV RNA(+) were stained by FISH (red). **(D)** Quantification of percentage of core/NS4B or core/NS5A structures co-localizing with RNA(+) (left), of RNA(+) co-localizing with core (middle), or of RNA(+) co-localizing with NS4B/NS5A structures. Scale bars of panels and zooms from squared area represent 10μm and 2μm, respectively. For each condition, over 20 cells were quantified.

We then investigated the sites of HCV assembly, which are represented by ER-derived areas where structural and non-structural viral proteins co-cluster with HCV RNA [66]. As compared to parental virus, we did not find significantly altered clustering of core, E2, NS4B, and NS5A for the Jc1 HAHALp7 virus (Fig 8A and 8B), suggesting identical rates of early assembly events. Likewise, we did not observe strong differences in the number of core structures co-localizing at assembly sites with HCV positive strand RNA for wt *vs*. mutant viruses (Fig 8C and 8D). Altogether, these results indicated that the initiation of early assembly events, *i*.*e*., allowing clustering of the HCV structural components at the ER membrane, were not impaired by p7 ATMI mutations.

**Fig 9:**
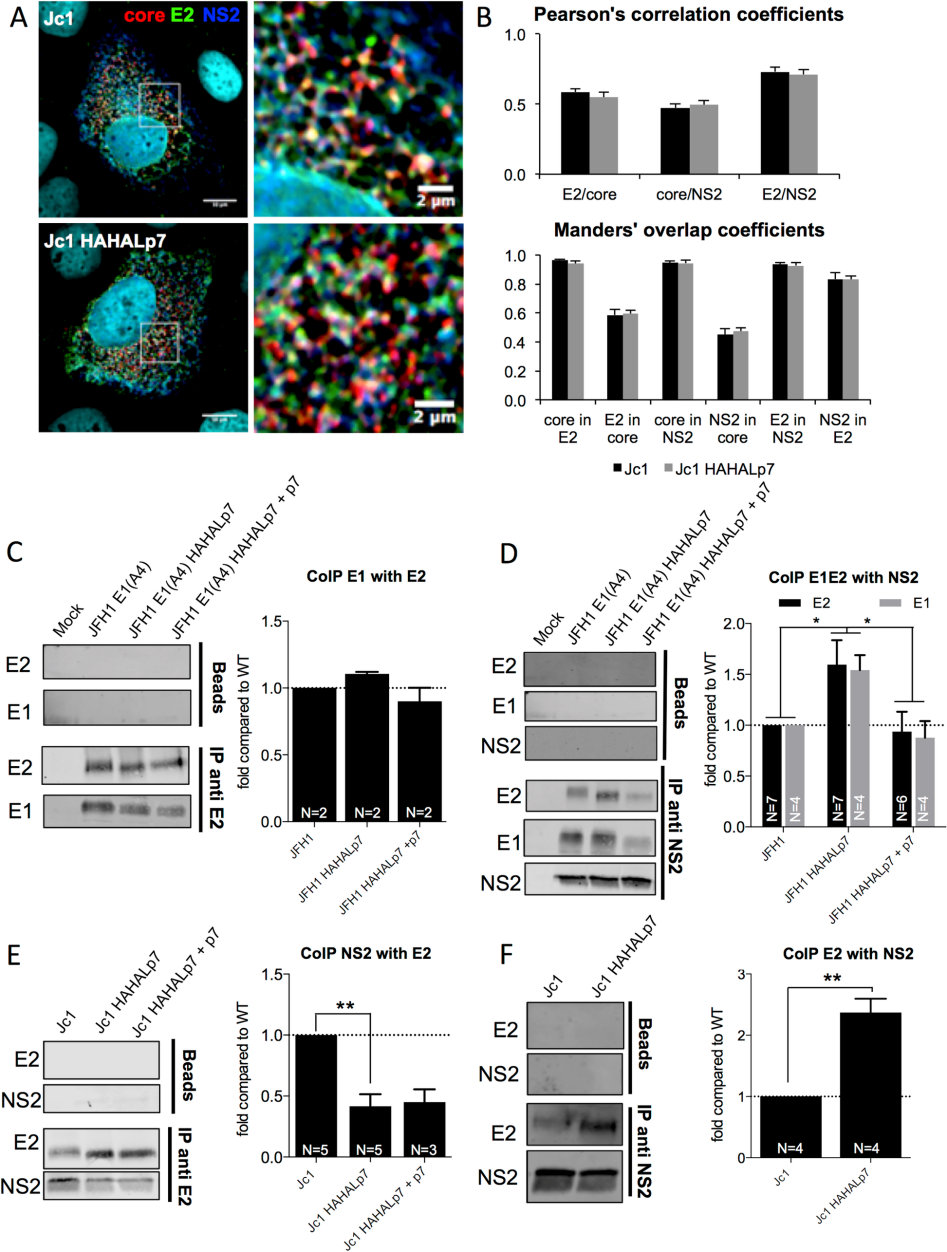
p7 ATMI mutant viruses alter NS2 and E2 association. Huh7.5 cells expressing RNAs from parental *vs*. Jc1 HAHALp7 or JFH1 HAHALp7 mutant viruses expressed alone or with wild-type p7 were analyzed at 72h. For immuno-precipitation, cell lysates were incubated with protein A/G agarose beads after pre-treatment, or not (beads), with NS2 or E2 antibodies. Immuno-precipitated complexes were eluted and analyzed by quantitative western blot. (**A**) Confocal microscopy of Huh7.5 cells expressing Jc1 or Jc1 HAHALp7 viruses and stained for HCV core (red), E2 (green) and NS2 (blue). (**B**) Co-localization analysis from (**A**), displaying the Pearson’s correlation coefficients and the Manders’ overlap coefficients, as indicated. **(C)** Representative western blot of E1 proteins co-immuno-precipitated with E2 antibodies (left). The levels of co-immuno-precipitated E1 proteins are normalized to the amount of immuno-precipitated E2 from JFH1 or JFH1 HAHALp7 virus-expressing cells (right). (**D**) Representative western blot of E1 and E2 proteins co-immuno-precipitated with NS2 antibodies (left). The levels of co-immuno-precipitated E1 and E2 proteins are normalized to the amount of immuno-precipitated NS2 in JFH1 or JFH1 HAHALp7 virus-expressing cells (right). Note that we used the JFH1 E1(A4) and JFH1 E1(A4) HAHALp7 viruses to detect E1 in these CoIP experiments. (**E**) Representative western blot of NS2 proteins co-immuno-precipitated with E2 antibodies (left). The levels of co-immuno-precipitated NS2 proteins are normalized to the amount of immuno-precipitated E2 from Jc1 or Jc1 HAHALp7 virus-expressing cells (right) **(F)** Representative western blot of E2 proteins co-immuno-precipitated with NS2 antibodies (left). The levels of co-immuno-precipitated E2 proteins are normalized to the amount of immuno-precipitated NS2 in Jc1 or Jc1 HAHALp7 virus-expressing cells. Data represent mean values ± SEM. The values are displayed relative to co-immuno-precipitation assays from JFH1 or Jc1 virus-expressing cells. The numbers of experiments performed are indicated below the graphs.

Next, since the NS2 non-structural protein is thought to serve as a scaffold for gathering virion assembly components through its interaction with E1E2 [9, 13–16, 71] and since p7 regulates this association [9, 14, 39], we tested if our p7 ATMI mutants had impaired E1E2/NS2 association. Using confocal microscopy, we did not detect altered co-localization of core, E2 and NS2 (Fig 9A and 9B), further highlighting that the assembly sites of viral particles were not grossly changed. However, when we analyzed E1 and NS2 co-immuno-precipitation with E2 antibodies, although the E1/E2 association was unchanged (Fig 9C), we detected a 3–4 fold decreased association of E2 with NS2 for p7 ATMI mutant *vs*. wt viruses (Fig 9E; S10A Fig). These results indicated that, relative to wt virus, the higher amounts of intracellular HCV glycoproteins detected for the p7 ATMI mutant viruses (Fig 3B and 3C; S3 Fig; S7 Fig) were not associated to NS2. This implied that only a fraction of the up-regulated E2 levels interact with the NS2 assembly platform, and suggested that part of the pool of HCV glycoproteins not associated to NS2 or to assembly sites induce the formation of SVPs. Thus, we performed the reverse co-immuno-precipitation with NS2 antibodies to more directly address interactions between assembly proteins at assembly sites. Strikingly, when we analyzed E1 and E2 co-immuno-precipitation with NS2, we found *ca*. 2–3 fold increased E1E2 association to NS2 in cells expressing Jc1 (Fig 9F) or JFH1 (Fig 9D; S10B Fig) p7 ATMI mutant viruses, as compared to parental viruses. Furthermore, these altered interactions with NS2 were reversed upon ectopic expression of wt p7 (Fig 9D; S10B Fig). Thus, since ectopically-expressed p7 did not restore wt intracellular E2 expression (Fig 3C) and since NS2 expression was unchanged for the mutant virus compared to wt (Fig 3D), this indicated that p7 N-terminus modulates NS2 association with HCV glycoproteins.

**Fig 10:**
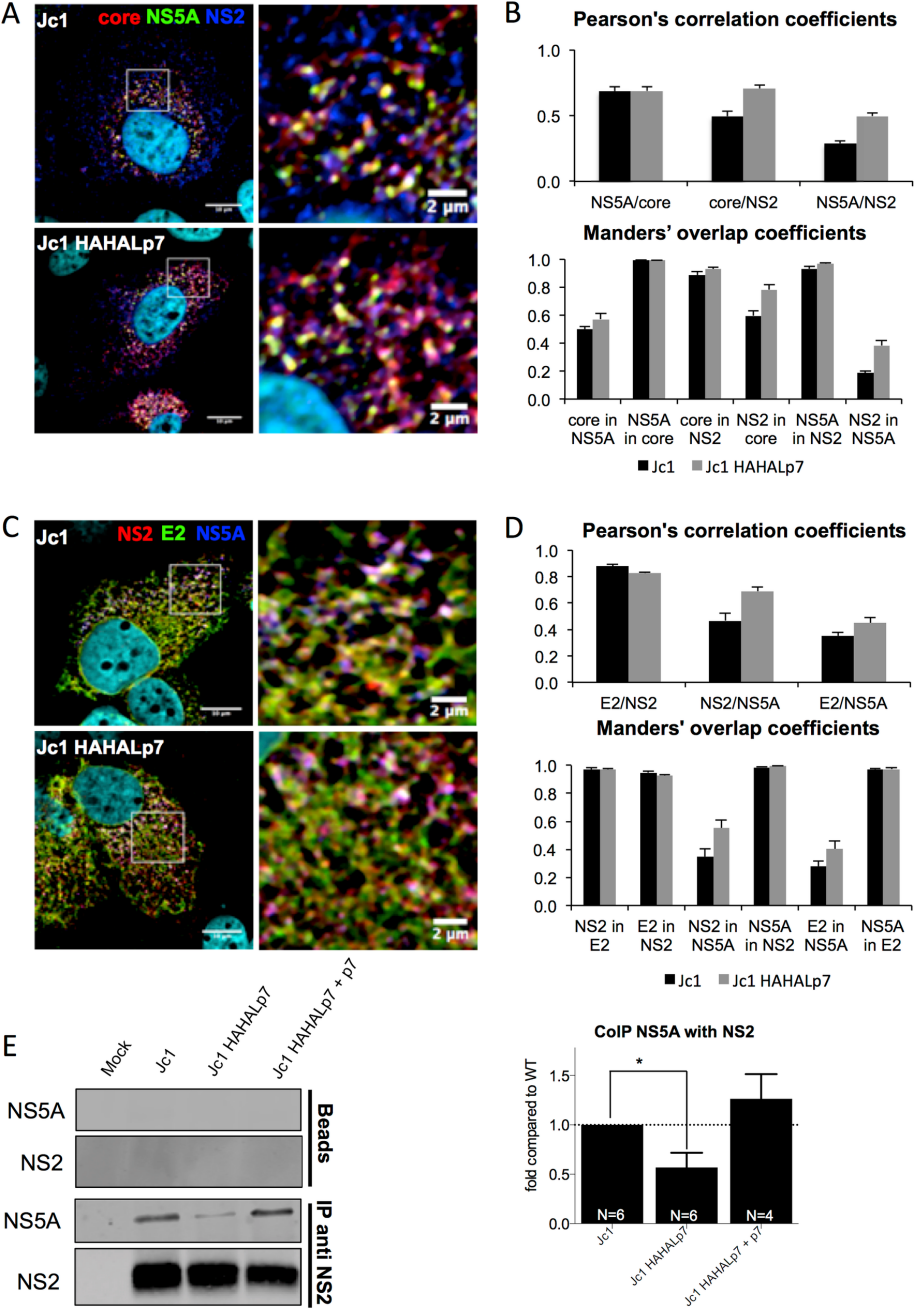
HCVcc mutants with modified p7 amino-terminus impair NS2 and NS5A association. Huh7.5 cells expressing RNAs from parental *vs*. Jc1 HAHALp7 mutant viruses expressed alone or with wild-type p7 were analyzed at 72h. (**A**) Confocal microscopy of Huh7.5 cells expressing Jc1 or Jc1 HAHALp7 viruses and stained for HCV core (red), NS5A (green) and NS2 (blue). (**B**) Co-localization analysis from (**A**), displaying the Pearson’s correlation coefficients and the Manders’ overlap coefficients, as indicated. (**C**) Confocal microscopy of Huh7.5 cells expressing Jc1 or Jc1 HAHALp7 viruses and stained for HCV NS2 (red), E2 (green) and NS5A (blue). (**D**) Co-localization analysis from (**C**), displaying the Pearson’s correlation coefficients and the Manders’ overlap coefficients, as indicated. (**E**) Cell lysates were incubated with protein A/G agarose beads after pre-treatment, or not (beads), with NS2 antibodies. Immuno-precipitated complexes were eluted and analyzed by quantitative western blot (see representative western blot to the left). The graphs show the levels of NS5A proteins co-immuno-precipitated by NS2 antibodies normalized to the amount of immuno-precipitated NS2 in Jc1 or Jc1 HAHALp7 virus-expressing cells. The values are displayed relative to association of NS5A to NS2 immuno-precipitated from Jc1 virus-expressing cells. Data represent mean values ± SEM. The numbers of experiments performed are indicated below the graphs.

Finally, since NS5A interacts with HCV RNA [72, 73] and core [18, 19] as well as with NS2 [13, 15], which likely transfers core and HCV RNA to assembly sites or to nascent viral particles [18–20], we investigated NS2 association with NS5A. No significantly altered co-localization of core, E2, NS2 and NS5A could be detected while comparing mutant *vs*. parental viruses (Fig 10A–10D), again underscoring the proximity of these different factors at assembly sites that appeared unaltered qualitatively. However, as compared to parental virus, we found a reduced NS5A co-immuno-precipitation with NS2 in cells expressing the Jc1 HAHALp7 or other p7 ATMI mutant viruses (Fig 10E; S9C Fig), which was restored upon co-expression with wt p7 (Fig 10E).

Altogether, these results suggested that the p7 amino-terminus determines the fine-tuning of the interactions between HCV glycoproteins, NS5A and NS2 required for envelopment of viral particles at assembly platforms.

## Discussion

We report here novel functions of the HCV p7 viroporin, which appears to modulate i) the cell secretory pathway, ii) the assembly and proportion of different secreted HCV-derived particle forms, including SVPs, partially enveloped core particles and infectious virions, and iii) the specific infectivity of the latter type of particles. Overall, our results provide novel insights in the properties of p7, particularly regarding the role of its junction segment with E2 and its retarded cleavage from E2. This distinguishes different functions of p7 between those that only depend on delayed E2p7 cleavage from those that reveal the role played by the p7 amino-terminus itself in envelopment and production of infectious viral particles.

### E2p7 cleavage rate regulates E2 intracellular levels

Because of their intrinsic capacity to be routed to the cell surface, owing to their localization in the cell secretory pathway, the traffic and distribution of envelope glycoproteins need to be controlled, by *e*.*g*., retention in specific intracellular compartments, in order to avoid immune detection of infected cells. Moreover, as these glycoproteins are synthesized in the ER lumen, counteracting ER stress activation in infected cells is important to avoid subsequent cell death. Since HCV proteins are expressed from a polyprotein, implying that all proteins are initially expressed at the same rate, its well-ordered cleavage may regulate the stability and functions of some proteins, such as for E2 and p7 that coexist with a E2p7 precursor (Fig 2) of ill-defined functions [7–11]. Here, by designing mutants at E2-p7 junction, we show that the augmentation of E2p7 cleavage, which is mediated by signal peptidase [7, 52], induced an up-regulation of the levels of E2 in infected cells, by *ca*. 4–5 fold.

This original phenotype is not caused by increased rates of replication or translation of such mutant viruses, judging from similar levels of viral RNAs or of non-structural proteins, respectively, for mutant *vs*. wt viruses. That both short peptide extensions and single alanine insertion before p7 structure induced E2p7 increased cleavage and E2 up-regulation at similar levels (Figs 2C and 3C; S3A Fig) argued against the possibility that p7 N-terminal modifications could *per se* change E2 expression. Consistently, co-expression of wt p7 with these mutant viruses did not restore wt E2 intracellular levels (Fig 3C).

Different possibilities may explain how E2p7 processing could modulate E2 expression. On the one hand, liberated E2 could be stabilized by its partners, such as *e*.*g*., E1 [74, 75] or SPCS1 [71]. On the other hand, E2 and E2p7 could activate or block different degradation pathways, such as autophagy or proteasome/lysosome. Indeed, HCV glycoproteins are known to be activators of the unfolded protein response (UPR) in HCV-infected cells [76]. Finally, it is also possible that the amounts of free p7, which are likely higher for the p7 ATMI mutant viruses, may regulate these degradation pathways. In support of these assumptions, a previous study [77] indicated that a mutation of p7 reported to block cleavage between E2 and p7 (p7-R(K)GR33-35AAA) [9, 11, 13] induced E2 degradation. Likewise, mutants abrogating E2p7 cleavage, such as E2-A367R (Fig 2) as well as p7-A1W, p7-E3W, p7-K4W, p7-A10W or p7-S12W [10], exhibited poor E2p7 expression despite wt rates of replication, relative to parental viruses. Further studies will be necessary to clarify this issue.

### Processing of E2p7 modulates E2 targeting to NS2 assembly platform

Regulation of the intracellular quantities of surface glycoproteins as well as their recruitment at virion assembly sites is crucial for production of infectious particles, which require optimal E1E2 incorporation levels to mediate entry into cells. Several cellular factors promoting the different steps of HCV assembly have been identified [3] and include factors allowing initial core and NS5A targeting at the LDs [78–82], HCV particle assembly [71, 83], or fission of enveloped nucleocapsids [84, 85]. As for viral factors, NS2 gathers assembly components at ER-localized sites near LDs and replication complexes [13, 15, 16, 86], at detergent-resistant membranes (DRM) areas [9, 39]. Specifically, NS2 interaction with E2 and E1 as well as with other viral factors—p7, NS3 and NS5A, is believed to be key for virion biogenesis [9, 13–15, 71, 84]. Yet, independent of this capture mechanism, E1E2 glycoproteins have the intrinsic capacity to induce SVP formation (Fig 1) [24], which implies a competition for their recruitment at the assembly sites of infectious particles. Noteworthy, E2/NS2 interaction depends on SPCS1, one of the 5 subunits of signal peptidase, and abrogation of E2/NS2/SPCS1 triple interaction *via* SPCS1 silencing markedly reduced HCV assembly [71].

Our co-IP assays indicated that increasing E2p7 cleavage, concomitantly with augmented E2 expression and SVP formation, stimulated the interaction between E1E2 and NS2 (Fig 9D and 9F; S10B Fig). A first, simple possibility to explain this stronger E1E2/NS2 association may involve the increase of E2 intracellular expression, which, incidentally, would lead to greater opportunities for E2/NS2 association. A second possibility could involve either a concentration of SPCS1 at the vicinity of E2 and NS2, as a result of SPCS1 recruitment by the signal peptidase complex during E2p7 and p7NS2 processing, or, alternatively, of a preferential interaction of cleaved, liberated E2 with SPCS1 and hence, with NS2. However, both possibilities would be difficult to reconcile with the finding that wt p7 co-expression, which did not restore wt E2 intracellular levels (Fig 3C), restored normal levels of E1E2/NS2 interaction (Fig 9D and 9F; S10B Fig). A third possibility is that the alteration of p7 N-terminus in our E2-p7 junction mutants may affect NS2 capacity to interact with some of its other partners, as discussed below. Unexpectedly, our results indicated that the increased E1E2/NS2 interaction correlated with reduced formation of infectious particles, in agreement with lowered secretion of nucleocapsids (Fig 5C). In this respect, it is likely that a loss of viral particle formation would translate in an increase of E2 density on NS2 platforms (Fig 9D and 9F) because E2 would not be consummated in assembled and released virions.

### Processing of E2p7 uncovers novel p7 assembly functions

Our results indicate that the p7 N-terminus also determines HCV infectivity by controlling the secretion of enveloped *vs*. naked/partially enveloped core particles (Fig 6B and 6D). This is in agreement with a previous report showing that p7 regulates the envelopment of nascent viral particles [40]. A recent study indicated that the first helix of p7 harbors a key determinant of HCV infectivity (*e*.*g*., V6, H9, S12), as underscored by mutagenesis of these residues pointing toward the p7 channel pore [10]. Intriguingly, we reveal here for the first time, a novel determinant at the extreme amino-terminal end of p7, *i*.*e*., before its first helix (S1 Fig), that strongly modulates infectivity (Fig 2) and the relative amounts of enveloped *vs*. non/partially-enveloped core particles (Fig 6B). Furthermore, we demonstrate that changes in this amino-terminal p7 determinant (p7 ATMI mutants), *via e*.*g*., short peptide extensions, single amino-acid insertions or substitutions that did not alter p7 structure (S1 Fig), induced a stronger reduction of infectivity (Fig 2) than of secretion of enveloped core particles (Fig 6D), resulting in reduced specific infectivity of the mutants, by 4- to over 50-fold depending on ATMI mutant types (Fig 4G–4I; S5 Fig.

Envelopment of viral particles pertains to a series of events that likely occur rapidly once two components, *i*.*e*., surface glycoproteins and nucleocapsids, encounter at assembly sites following mobilization from their respective storage pools, *i*.*e*., respectively, NS2 platforms apposed to LDs [9, 39] and LDs/replication complexes [12]. Such events are difficult to catch experimentally, because they are transient by nature as they lead to quick release of assembled viral particle from such assembly sites within the ER lumen. How HCV core and RNA are transferred from LD surface to ER assembly sites to initiate the release of infectious, enveloped viral particles remains poorly defined [3, 87], although it involves concerted actions of p7 and NS2 [17] and of NS5A [18, 20]. Accordingly, previously described assembly-defective mutants, such as p7-KR33/35QQ and core-C69-72A [40], ∆p7 [65] or ∆E1E2 and NS5A-∆2328–2435 [20], display strong core-LD accumulation, which correlates with their loss of infectivity. Strikingly, in contrast to these previous assembly mutants but similar to parental viruses, our Jc1 p7 ATMI mutants readily targeted core at the ER membrane (S8 Fig) despite reduced infectivity and did not significantly alter the co-clustering of structural and non-structural proteins with HCV RNA (Figs 8–10), both of which events previously shown to be critical for achieving efficient assembly [17, 65, 66]. Moreover, as shown by others, p7 regulates NS2 subcellular localization at punctate sites near LDs and its association with DRMs along with other viral proteins, including core, E2, and NS3 [9, 16, 39, 86]; yet, while other assembly-defective virus mutants, such as the p7-KR33/35QQ and p7-KR33/35AA in these previous reports, disrupted NS2 localization and/or E2/NS2 association, the p7 ATMI mutants displayed increased E1E2/NS2 interaction, compared to parental viruses. Along with the finding that our mutants exhibited wt capacity to slow down the cell secretory pathway (Fig 1E), this underscores that the ATMI class of p7 mutants retains most p7 properties and inhibits viral assembly though a novel mechanism.

**Fig 11:**
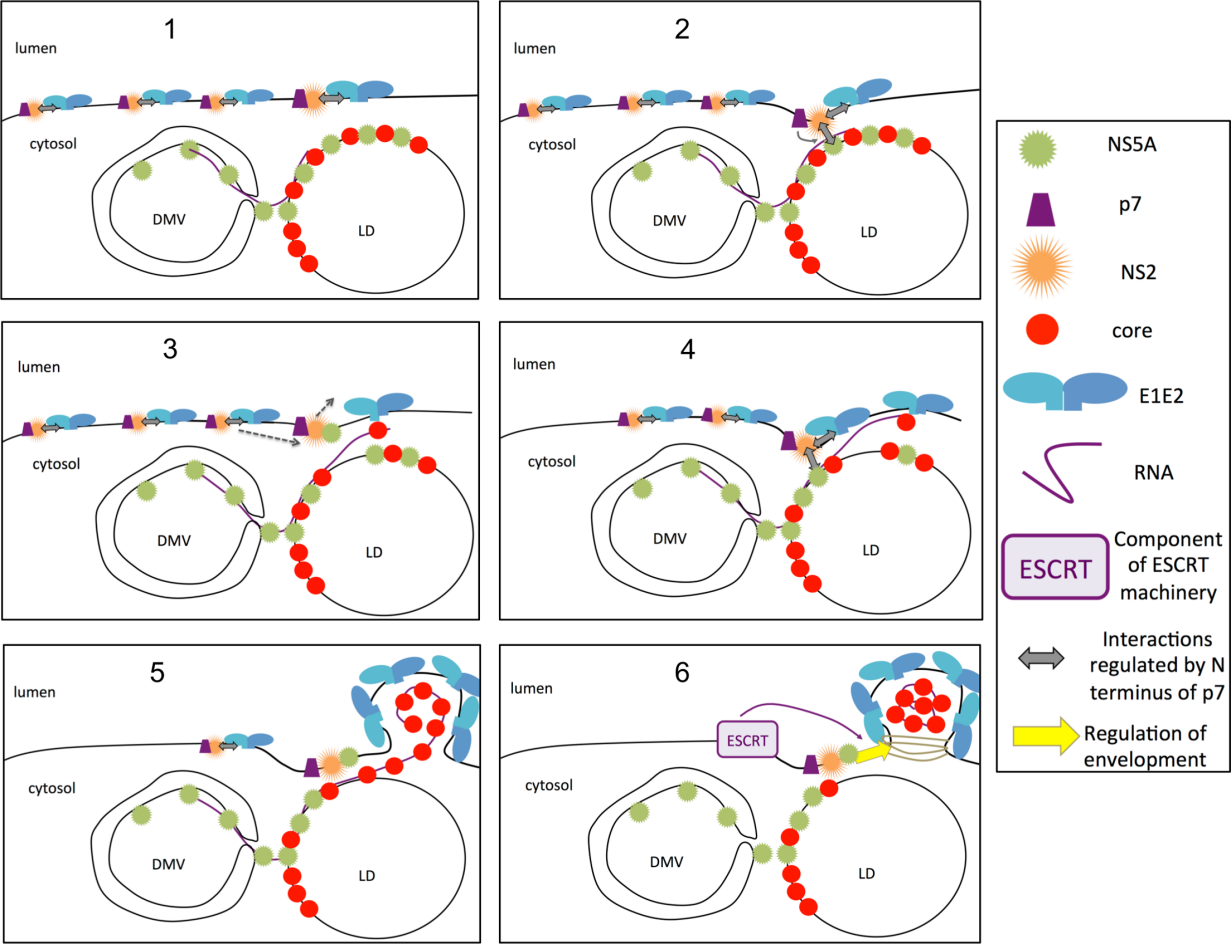
Model of p7 role in assembly and envelopment of HCV particles. (**1**) HCV assembly takes place at the ER membrane close to HCV replication complexes-containing double membrane vesicles (DMV) and to cytosolic lipid droplets (LD) harboring core and NS5A at their surface. Upon translation of the HCV genome, E1E2p7NS2 complexes form and accumulate at the ER membrane. (**2**) p7, *via* its N-terminal extremity, regulates (thin grey arrow) the interaction of NS2 with NS5A. (**3**) This interaction may allow the release of E1E2 from E1E2p7NS2 complexes at the assembly site of nascent viral particles on the one hand and co-recruitment of core and RNA to E1E2 on the other hand. Subsequently, the E1E2-free p7NS2NS5A complex leaves whereas a new E1E2p7NS2 complex reaches the assembly area (dotted arrows). (**4**) The process is reiterated with p7 from incoming E1E2p7NS2 complexes regulating the encountering of NS2 and NS5A, which leads to the further release of E1E2 and core/RNA that accumulate at the assembly site. (**5**) The process is repeated until the formation of a viral particle that buds in the ER lumen. (**6**) The NS2NS5A complex, regulated by p7, may recruit ESCRT components to induce scission of the nascent particle until full envelopment and egress.

Our results imply an envelopment defect caused by inadequate mobilization and/or transfer of core and RNA at E1E2-containing NS2 assembly platforms. This is reflected by our findings that such p7 ATMI mutants exhibited altered E1E2/NS2 and NS2/NS5A interactions (Figs 9 and 10) but also that failure to mediate correct particle envelopment resulted in secretion of partially enveloped, proteinase K-sensitive core particles (Fig 6B). Since co-expression of our mutant viruses with wt p7 restored the above alterations to almost normal levels, this questions about the role of p7 N-terminus in this mechanism and raises the possibility that it regulates core and RNA transfer to assembly sites and/or to assembling viral particles (Fig 11). Interestingly, a previous report suggested that p7 genetically interacts with some regions of NS2 as well as of NS5A [88], strengthening the notion of a functional dialog between p7, NS2 and NS5A. Our findings that the N-terminus of HA-tagged p7 points towards the cytosol (Fig 7) support the likelihood that it mediates critical interactions with cytosolic factors promoting assembly. A possibility is that modifications of p7 N-terminal surface, before the first p7 helix (S1 Fig), disrupted such interactions (Fig 11). In this respect, co-localization and association of NS2 with NS5A, which is decreased upon p7 deletion or alterations [13, 16], is thought as a crucial event mediating core/RNA transfer to assembly sites [18, 20, 66]. Thus, since our mutant viruses did not display altered co-localization of these assembly factors, it is likely that NS2 complexes formed with p7 ATMI mutants failed to efficiently mediate the encountering of nucleocapsids with NS2-bound viral surface components and/or to induce the release of fully enveloped, infectious viral particles. Why such failure resulted in a proportional release of partially enveloped core particles (Fig 6B *vs*. Fig 6D), which could be similar to those that have been detected in the serum of infected patients [28], is intriguing. It raises the possibility that the transfer of core and RNA to E1E2 glycoproteins liberated from NS2 complexes at assembly sites is associated to the recruitment of a mechanism that closes nascent particles (Fig 11). Such a mechanism, which likely involves components of the ESCRT pathway, as previously described for HCV [84, 85], could be disrupted by p7 ATMI mutants is such a way that, rather than being correctly closed, the budding membrane capsule would detach, allowing escape of imperfectly enveloped nucleocapsids in the ER lumen, which could explain their reduced specific infectivity. Indeed, as p7 ATMI mutants impair the interaction between NS2 and NS5A, this may prevent the recruitment of HRS, an ESCRT-0 component that interacts with p7, NS2 and NS5A [84] and subsequently, of all ESCRT components required for correct envelopment.

### Free p7 slows down the cell secretory pathway

Our data indicate that the regulation of the amounts of free p7 could modulate the production of viral particles. Indeed, we found that p7, which localizes at the ER (Fig 7) [89] slows down the cell secretory pathway in a dose-dependent manner, likely at the stage of ER-Golgi transport (Fig 1). While this property is not intuitive, given that HCV, like other *Flaviviridae*, is thought to exit the cells through the secretion pathway [90], this could either induce the concentration of its glycoproteins at virion assembly sites in the ER lumen through their active retention or reflect the cooptation of another pathway of secretion for HCV particles [22, 23]. Furthermore, as HCV glycoproteins can be secreted as SVPs independently of other viral proteins (Fig 1G) [24], this feedback loop may ensure that excess glycoproteins, arising from their release upon E2p7 cleavage, could be appropriately controlled so as to prevent activation of immune responses. Additionally, as indicated by the delayed transport of VSV-Gts used as a model cargo (Fig 1B–1F), it is possible that p7 expression could alter the secretion of cellular proteins such as, *e*.*g*., immune effectors, as shown for viroporins of other viruses [43, 91].

The mechanism used by p7 to slow down the secretion of glycoproteins needs further investigation. Viroporins of alternative viruses have previously been involved in modulation of the secretory pathway, though through a variety of mechanisms [38]. For example, the M2 protein from influenza virus has a direct effect on late steps of plasma membrane delivery by delaying late Golgi transport, which indirectly affects the efficiency of earlier transport steps by altering the ionic content of the Golgi apparatus and the endosomes [92, 93]. Alternatively, Coxsackievirus 2B proteins modify ER membranes, which inhibits protein processing and sorting by decreasing calcium homeostasis in ER and Golgi [43]. Likewise, p7 can change ionic gradients in both reconstituted membrane assays *in vitro* [30, 44–47] and *in cellulo* [41, 42], which could affect anterograde transport and/or modify intracellular compartments.

In conclusion, our report underscores the function of E2p7 delayed processing in modulating i) the intracellular E2 levels, ii) the retention of E2 through the slowing down of the secretion pathway, and iii) the unmasking of functions of p7 amino-terminus in assembly and envelopment.

## Materials and methods

### Cell culture and reagents

Huh7.5 cells (kind gift of C Rice, Rockefeller University, New York, USA) and 293T kidney (ATCC CRL-1573) cells were grown in Dulbecco’s modified minimal essential medium (DMEM, Invitrogen, France) supplemented with 100U/ml of penicillin, 100μg/ml of streptomycin, and 10% fetal bovine serum.

### Plasmids and constructs

pFK-JFH1wt_dg, pFK-JFH1/J6/C-846_dg plasmids encoding full-length JFH1 and Jc1 HCV [94] were kind gifts from R Bartenschlager (Heidelberg University, Germany). pFK-JFH1J6XbaIC-846HAHA-L-p7_dg encoding a Jc1 virus with the HAHALp7 linker peptide between E2 and p7 [56] was kindly provided by T Pietschmann (Twincore, Germany). JFH1 virus-derived constructs encoding the p7-T2, p7-L2S, Ap7 and ASGGSp7, HAHALp7, and E2-A367R mutants were derived from the pFK-JFH1wt_dg plasmid. The E1 glycoprotein was also point-mutated in the pFK-JFH1wt_dg and pFK-JFH1 HAHALp7 constructs to introduce the A4 epitope, resulting in plasmids encoding JFH1 E1(A4) and JFH1 E1(A4) HAHALp7 viruses, respectively [57]. Constructs were created by PCR mutagenesis (oligonucleotide sequences are available upon request).

The plasmid pEGFP-N3-VSV-Gts was a kind gift from K Konan (Albany Medical College, USA). The plasmids encoding noSPp7 (JFH1), ΔE2p7 (JFH1), ΔCp7 (JFH1), ΔCp7 (H77), ΔE2p7 (J6), ΔE2HAHALp7 (JFH1), and noSPHAHALp7 (JFH1) allow individual expression of wt, variant or mutant p7 under different signal peptide configurations. The plasmids pTG 13077-HCV-ΔC-E1-E2-J6, pTG 13077-HCV-ΔC-E1-E2-JFH1 and pTG 13077-HCV-ΔC-E1-E2-p7-JFH1 contain retroviral vector genomes encoding E1E2 and/or E1E2p7 proteins from J6 and JFH1 viruses.

All constructs were expressed in Huh7.5 cells using procedures reported before [17].

### Antibodies

Mouse anti-actin (clone AC74, Sigma-Aldrich), mouse anti-E1 A4 (kind gift from HB Greenberg), rat anti-HA (clone 3F10, Roche), mouse anti core C7-50 (Thermo Fisher Scientific), rat anti-E2 clone 3/11 (kind gift from J McKeating), mouse anti-NS2 6H6 and mouse anti-NS5A 9E10 (kind gift from C Rice), rabbit anti-NS2 (kind gift from B Lindenbach), human anti-E2 AR3A (kind gift from M Law), mouse anti-GFP (Roche), anti-VSV-G 41A1, mouse anti-E2 antibody AP33 (kind gift from A Patel) were used according to the providers’ instructions.

### VSV-Gts analysis

Huh7.5 cells were seeded 16h prior to transfection with pEGFP-N3-VSV-Gts and p7-encoding plasmids using GeneJammer transfection reagent (Agilent). Medium was changed 4h post-transfection and cells were incubated overnight at 40°C. 24h post-transfection, cells were chased at 32°C. For western blot analysis, cells were lysed at indicated time points in wells cooled on ice before clarification and western blot analysis. For flow cytometry analysis, cells were harvested and put in suspension at 32°C. At indicated time points, cells were fixed with 3% paraformaldehyde.

### Purification of soluble E2 (JFH1)

The plasmid popol-ΔE1sE2 (JFH1)-H6 (kind gift from Epixis SA) encoding soluble E2 (JFH1) with a 6xHis tag was used to purify E2 in order to assess the sensitivity of E2 quantifications by western blots. 293T cells grown in 10 cm-plates were transfected with 15μg of popol-ΔE1sE2 (JFH1)-H6. 16h post-transfection, the medium was replaced by OptiMEM. 24h and 48h later, supernatant was harvested and purified using a HisTrap column. Fractions were pooled and then dialyzed. A sample was analyzed by SDS-PAGE. Concentration of sE2 was obtained by measurement of OD at 280nm and purity was analyzed by LC-MS/MS.

### Production of lentivirals vectors

HEK293T cells were seeded 24h prior to transfection with VSV-G plasmid, pTG-5349 packaging plasmid, and either pTG 13077-HCV-ΔC-E1-E2-JFH1, pTG 13077-HCV-ΔC-E1-E2-J6 or pTG 13077-HCV-ΔC-E1-E2-p7-JFH1 plasmids using calcium phosphate precipitation. Medium was replaced 16h post-transfection. Vector supernatants were harvested 24h later, filtered through a 0.45 μm filter, and were titrated by flow cytometry using AP33 antibody against E2.

### Production of SVPs

Lentiviral vectors were used to transduce Huh7.5 cells (MOI = 2). 72h post-transduction, cell supernatants were centrifuged at 25,000 rpm for 4h at 4°C using SW41 rotor and Optima L-90 centrifuge (Beckman). Pellets were suspended in PBS prior to use for western blot analysis.

For gradient analysis, 1 ml of supernatant concentrated 40x by Vivaspin columns (MW cut-off 100-kDa (Sartorius)) was loaded on iodixanol density gradients. 12 fractions were collected from the top and used for refractive index measurement and precipitation of proteins before western blot analysis.

### Production of HCVcc particles and quantifications of virion components

Methods for *in vitro* transcription of HCV RNA and its electroporation into Huh-7.5 cells have been described [17, 61]. When p7 was co-expressed with viral RNA, 2μg of plasmid DNA encoding p7 or control DNA were co-electroporated with 10μg of viral RNA.

To determine the percentage of HCV-positive producer cells following electroporation, cells were fixed and stained using Cytofix/Cytoperm (BD) according to manufacturer's instructions. NS5A staining was achieved with 9E10 antibody (kind gift from C. Rice, Rockefeller University, New York, USA) and cells were analyzed using MacsQuant VYB (Milteny Biotech).

Electroporated cells were counted and 100,000 cells were lysed in lysis buffer (20 mM Tris [pH 7.5], 1% Triton X-100, 0.05% sodium dodecyl sulfate, 150 nM NaCL, 5‰ Na deoxycholate) supplemented with protease/phosphatase inhibitor cocktail (Roche) and clarified from the nuclei by centrifugation at 13,000×*g* for 10 min at 4°C for quantitative western blot analysis (see below).

HCV core protein was also quantified by CMIA—Chemiluminescent Microparticle ImmunoAssay (Architect, Abott). The extracellular E2 protein was quantified by Western Blot after precipitation of E2-containing cell supernatants with Galanthus Nivalis lectins (GNA) bound to agarose beads (Vector Laboratories). The extracellular HCV RNAs were quantified as described previously [61]. Infectivity titers were determined as focus-forming units per milliliter [17]. Serial dilutions of supernatants were used to infect Huh7.5 cells and focus-forming units were determined 3 days post-infection by counting NS5A-immunostained foci. For determining intracellular infectivity, electroporated cells were washed with PBS, harvested with Versene and centrifuged for 4 min at 400xg. Cell pellets were suspended in medium and subjected to 4 cycles of freeze and thaw, using liquid nitrogen.

For purification of particles, supernatants were harvested and filtered through a 0.45μm filter and centrifuged at 25,000 rpm for 1h45 at 4°C with a SW41 rotor and Optima L-90 centrifuge (Beckman). Pellets were resuspended in PBS prior to use for western blot to quantify E2 or for quantification of core and RNAs.

### Proteinase K digestion assays

Viral supernatants were i) left untreated, ii) treated with Proteinase K (PK, 50 μg/mL) in 10x PK buffer as described in [84] for 1h on ice, or iii) pre-treated with Triton X-100 5min at room temperature prior to treatment with PK. PK activity was stopped by adding 10 mM PMSF and protease inhibitors cocktail (Roche). The core protein was quantified with CMIA.

### Iodixanol density gradient of HCVcc particles

1mL of viral supernatant was loaded on top of a 3–40% continuous iodixanol gradient (Optiprep, Axis Shield). Gradients were centrifuged for 16h at 4°C in Optima L-90 centrifuge (Beckman). 16 fractions of 750 μl were collected from the top and used for refractive index measurement infectivity titration, core quantification and RNA quantification, as described above. For E2 protein analysis, HCV particles were produced in OptiMEM (Invitrogen) and concentrated 40x by Vivaspin molecular weight cutoff 100-kDa columns (Sartorius). 1 ml of concentrated virus suspension was loaded on density gradients. 12 fractions were collected from the top and used for refractive index measurement, titration, core quantification and RNA quantification, as described above. The remaining volumes of fractions were used for protein precipitation with 4 volumes of acidified acetone/methanol buffer and left at -20°C overnight. Proteins were pelleted at 16,000xg for 15min and dried before resuspension in lysate buffer, denaturation in Laemmli buffer, and Western Blot analysis.

### Deglycosylation with endoglycosidase H

Endoglycosidase Hf (Endo-Hf; NEB) treatment was performed according to the manufacturer's recommendations. Briefly, protein samples were mixed to denaturing glycoprotein buffer and heated at 100°C for 5 min. Subsequently, 1,000 units of Endo-Hf were added to samples in a final volume of 25 μl and the reaction mixtures were incubated for 1 h at 37°C, before western blot analysis.

### Western blot analysis

Proteins obtained in total lysates or after digestion or immunoprecipitation, were denatured in Laemmli buffer at 95°C for 5min and were separated by sodium dodecyl sulfate polyacrylamide gel electrophoresis, then transferred to nitrocellulose membrane and revealed with specific primary antibodies, followed by the addition of IRdye secondary antibodies (Li-Cor Biosciences), followed by imaging with an Odyssey infrared imaging system CLx (Li-Cor Biosciences).

### Co-immuno-precipitation assays

For NS2/E2 interaction, 1 million electroporated cells were lysed with buffer (50 mM Tris-Cl (pH 7.5), 150 mM NaCl, 1% Nonidet P-40, 1% sodium deoxycholate, and 0.1% SDS). Lysates were cleared by centrifugation at 16,000xg for 10 min at 4°C and were incubated overnight at 4°C with AR3A antibody against HCV E2 or with rabbit NS2 antibody. Protein A/G-coated agarose beads were added to samples for 2h at room temperature. Immune complexes were then washed and eluted with Laemmli buffer for 5 min at 95°C before western blot analysis.

For NS2/NS5A interaction, 1 million electroporated cells were cross-linked with 1mM dithiobis(succinimidyl propionate) (DSP) (ThermoFisher) 30min at room temperature. Tris (pH 7.5) was added up to 200 mM to quench unreacted DSP. Cells were resuspended in lysis buffer (50mM Tris pH 7.4, 150mM NaCl, 1mM EDTA, 0.5% n-dodecyl-β-maltoside) and treated as for NS2/E2 interaction with incubation with rabbit NS2 antibody.

### Immuno-fluorescence (IF) and confocal microscopy imaging

Experimental procedures were previously described [66]. Briefly, Huh7.5 cells grown on glass coverslips and were infected at MOI of 0.2. 72h post-infection, cells were washed with PBS, fixed with 3% paraformaldehyde in PBS for 15min, quenched with 50mM NH_4_Cl and permeabilized with 0.1% Triton X-100. Fixed cells were then incubated with primary antibodies in 1% BSA/PBS, washed and stained with the corresponding fluorescent Alexa-conjugated secondary antibody (Alexa-488, Alexa-555 and Alexa-647, Molecular Probes) in 1% BSA/PBS. LDs were stained with 10μg/mL Bodipy 493/503 (Molecular Probes) according to the manufacturer’s instructions. Cells were washed with PBS, stained for nuclei with Hoechst (Molecular Probes) and mounted with Mowiol 4–88 (Sigma-Aldrich) prior to image acquisition with LSM-710 (Zeiss) confocal microscope. When stated, the combined detection of HCV RNA by FISH and viral proteins was done as previously described [66].

For digitonin permeabilization, the staining procedure was the same except that cells were permeabilized with 5μg/ml Digitonin (Sigma-Aldrich) for 10 min. Cells permeabilized with Triton X-100 were acquired first; then, cells permeabilized with Digitonin were acquired in order to use the same laser settings.

### Image analysis and quantifications

Images were analyzed and quantified with the ImageJ software as previously described [66]. The Pearson’s and Manders’ correlation coefficients were calculated by using the JACoP plugin [95]. For the Digitonin *vs*. Triton permeabilization experiments, the relative fluorescence intensity of each channel was quantified by using the integrated density measurement of ImageJ software.

### Molecular modeling of p7 hexamers

Three-dimensional homology models of p7 hexamers and their mutants were constructed using the NMR/MD p7 model of Chandler and colleagues [36] and the NMR p7 structure of OuYang and colleagues [35] (PDB accession number 2M6X) as templates. Models of p7 were constructed with the Swiss-Model automated protein structure homology modeling server (http://www.expasy.org/spdbv/ [96]) using the HCV JFH1 strain p7 sequence as input. JFH1 p7 homology model derived from OuYang et al. was directly obtained as a hexamer by the automated procedure. For JFH1 p7 homology model derived from Chandler et al., raw amino-acid sequence of p7 from strain JFH1 was first loaded in Swiss-PdbViewer software [96] and fitted to the NMR/MD p7 hexamer model [36] before submission for model building to Swiss-Model using the SwissModel Project Mode. All p7 JFH1 mutants were constructed using the latter protocol, *i*.*e*., fitting of the raw amino-acid sequence of p7 mutants to wild type hexamer models from the JFH1 strain. Coordinates of homology models derived from the automated model building were used without further minimization or manual manipulation. For mutants Ap7 and ASGGSp7 exhibiting N-terminal extensions, additional residues were added manually assuming a random conformation and were minimized using Swiss-PDB Viewer tools.

### Statistical analysis

Significance values were calculated by applying the paired t-test using the GraphPad Prism 6 software (GraphPad Software, USA). For confocal analysis, a two-tailed, unpaired Mann-Whitney test was applied. P values under 0.05 were considered statistically significant and the following denotations were used: ****, P≤0.0001; ***, P≤0.001; **, P≤0.01; *, P≤0.05; ns (not significant), P>0.05.

## Supporting information

S1 Fig Molecular modeling of p7 ATMI mutant structures. Comparison of three-dimensional homology molecular models of p7 structures using the NMR/MD model in POPC [36] and the NMR model in DPC [35]. The first amino-acid positions are represented in blue, second positions in red, third positions in green. Single amino acid insertions are represented in orange whereas larger insertions are represented in violet.
(TIFF)

S2 Fig Transcomplementation of p7 ATMI mutant viruses with wt p7. Huh7.5 cells were electroporated with RNAs from parental or p7 ATMI mutant viruses, as indicated. At 72h post-electroporation, expression analyses were performed. (**A**) NS2, HAHALp7, E2, E2p7 as well as E2HAHALp7 (white arrows) and E2p7NS2 and E2HAHALp7NS2 (black arrows) precursors were revealed using anti-E2, anti-NS2 and HA antibodies, as indicated. (**B**) The infectivity levels of JFH1-derived p7 ATMI mutant viruses expressed alone (black bars) or with wild-type p7 (hatched bars) are represented.
(TIFF)

S3 Fig p7 ATMI mutant viruses accelerating E2p7 cleavage have increased expression levels of E2 and core proteins. Huh7.5 cells were electroporated with RNAs from parental or p7 ATMI mutant viruses, as indicated. At 72h post-electroporation, expression analyses were performed and determined by quantitative western blot. (**A**) Levels of intracellular E2 for the JFH1-derived p7 ATMI mutant viruses. (**B**) Levels of intracellular core for the same mutant viruses. Proteins in (**A**) and (**B**) were quantified and normalized after determining the proportion of HCV-positive virus producer cells and the amounts of cellular actin (see Fig 3A). (**C**) Huh7.5 cells were electroporated with RNAs from parental or Jc1 HAHALp7 mutant virus. At 72h post-electroporation, cells were treated with cycloheximide (100μg/mL) and brefeldin A (1μg/mL). At the indicated time points, cells were counted and the same amounts of cells were lysed. Levels of E2 were determined by quantitative Western blot. The values are displayed relative to expression of E2 and core in JFH1 HCVcc virus-electroporated cells (**A**, **B**) or relative to time 0h post-addition of the drugs (**C**). Data represent mean values ± SEM. The number of experiments performed are indicated below the graphs.
(TIFF)

S4 Fig p7 ATMI mutant viruses display increased secretion of E2 but decreased secretion of core proteins and RNA. Huh7.5 cells were electroporated with RNAs from parental or JFH1 mutant viruses expressed alone or with wild-type p7. At 72h post-electroporation, analyses were performed and normalized after determining the proportion of HCV-positive virus producer cells (see Fig 3A). (**A**) Levels of secreted E2 determined by quantitative western blot following GNA lectin pull down of cell supernatants. (**B**) Levels of secreted HCV RNAs as determined by RT-qPCR. (**C, D**) Levels of secreted core as determined by CMIA for JFH1 HAHALp7 or JFH1 p7-T2 mutant viruses alone or with WT p7 (**D**) and for other JFH1-derived p7 ATMI mutants (**C**). All values are displayed relative to expression of E2, core or RNA values determined in the supernatants of JFH1 virus-electroporated cells (**A-C**). Data represent mean values ± SEM. The number of experiments performed are indicated below the graphs.
(TIFF)

S5 Fig p7 ATMI mutant viruses exhibit decreased specific infectivity. Huh7.5 cells were electroporated with RNAs from parental or JFH1 mutant viruses expressed alone or with wild-type p7. At 72h post-electroporation, infectivity, and RNA and core secretion analyses were performed. (**A**) Specific infectivity relative to RNA amounts for all JFH1-derived p7 ATMI mutant viruses. (**B**) Specific infectivity relative to core amounts for all JFH1-derived p7 ATMI mutants. (**C**) Specific infectivity relative to core amounts for JFH1 HAHALp7 or JFH1 p7-T2 mutant viruses expressed alone or with wild-type p7. Values are displayed relative to expression of specific infectivity in the supernatants of JFH1-electroporated cells. Data represent mean values ± SEM. The number of experiments performed are indicated below the graphs.
(TIFF)

S6 Fig p7 ATMI mutant viruses have increased secretion of particle-associated E2 proteins. Huh7.5 cells were electroporated with RNAs from parental or JFH1 mutant viruses expressed alone or with wild-type p7. At 72h post-electroporation, quantitative western blot analyses were performed. (**A**) Level of E2 and E1 in pellet for the JFH1 HAHALp7 mutant virus relative to parental virus. (**B**) Level of E2 in pellets from ultracentrifuged cell supernatants for all p7 ATMI mutants. (**C**) Aliquots of supernatant from cells expressing JFH1 or JFH1 HAHALp7 viruses were incubated for 1hr with 1% Triton X-100 or left untreated before ultracentrifugation and analysis of E2 in the pellets by quantitative western blot. Proteins in (**A**) were quantified and normalized after determining the proportion of HCV-positive virus producer cells. Values are displayed relative to expression of E2 or E1 in the pellets of supernatants from JFH1 virus-electroporated cells. Data represent mean values ± SEM. The number of experiments performed are indicated below the graphs.
(TIFF)

S7 Fig Representative density gradient analysis of p7 ATMI mutant viruses in Jc1 or JFH1 HCVcc backbones. Huh7.5 cells were electroporated with RNAs from parental *vs*. Jc1 HAHALp7 (**A**), JFH1 HAHALp7 (**C**) viruses expressed alone or with wild-type p7 (**B**). At 72h post-electroporation supernatants were collected and layered on iodixanol buoyant density gradients. (**A**) Representative gradient profiles of Jc1 and Jc1 HAHALp7 viruses. The levels of E2, core, infectivity, and viral RNAs were quantified and normalized after determining the proportion of HCV-positive virus producer cells (see Fig 3A). (**B**) Representative gradient profiles of Jc1 and Jc1 HAHALp7 viruses expressed with or without wt p7. The levels of core and infectivity were quantified and normalized after determining the proportion of HCV-positive virus producer cells (see Fig 3A). (**C**) Representative gradient profiles of JFH1 and JFH1 HAHALp7. The core, E2 and E1 proteins and infectivity were measured in each fraction and expressed of percentages of the sum of fractions.
(TIFF)

S8 Fig p7 ATMI mutant viruses do not alter the intracellular localization of core. At 72h post-transfection or post-infection, cells were fixed and stained for HCV core (red), LD (green, left panels), calnexin (green, right panels) and nuclei (blue). (**A**) Confocal microscopy analysis of Huh7.5 cells transfected with constructs expressing J6 core alone or in combination with wt p7 or HAHALp7. (**B**) Confocal microscopy of Huh7.5 infected with Jc1 or Jc1 HAHALp7 viruses.
(TIFF)

S9 Fig Co-localization of p7 ATMI mutant and E2 with core protein. At 72h post-electroporation or transfection, cells were fixed, permeabilized with either Triton X-100 or Digitonin, as indicated, and stained for HCV core (red), HAHALp7 (green), E2 (green) and nuclei (grey). (**A**) Confocal microscopy analysis of Huh7.5 electroporated with JFH1 HAHALp7 virus RNAs. (**B**) Confocal microscopy analysis of Huh7.5 cells transfected with core-E1E2 and noSPHAHALp7 expression constructs. (**C**) Confocal microscopy analysis of Huh7.5 cells transfected with core-E1E2 and ∆E2HAHALp7 expression constructs. The relative fluorescence intensity of each channel was quantified by using ImageJ.
(PDF)

S10 Fig p7 ATMI mutants impair NS2 association with E2 and NS5A. Huh7.5 cells expressing RNAs from parental or JFH1-derived p7 ATMI mutant viruses expressed alone or with wild-type p7 were analyzed at 72h. **(A)** Levels of NS2 proteins co-immuno-precipitated by E2 antibodies normalized to the amount of immuno-precipitated E2 proteins. (**B**) Levels of E2 proteins co-immuno-precipitated by NS2 antibodies normalized to the amount of immuno-precipitated E2 proteins. **(C)** Levels of NS5A proteins co-immuno-precipitated by NS2 antibodies normalized to the amount of immuno-precipitated NS2 proteins. The values are displayed relative to co-immuno-precipitated E2, NS5A or NS2 in JFH1 virus-expressing cells. Data represent mean values ± SEM. The number of experiments performed are indicated below the graphs.
(TIFF)
