# Supplementary figures and images for: The amino-terminus of the hepatitis C virus (HCV) p7 viroporin and its cleavage from glycoprotein E2-p7 precursor determine specific infectivity and secretion levels of HCV particle types
Source: PLoS Pathog. 2017 Dec 18;13(12):e1006774. doi: 10.1371/journal.ppat.1006774 (PMC5749900; doi:10.1371/journal.ppat.1006774)

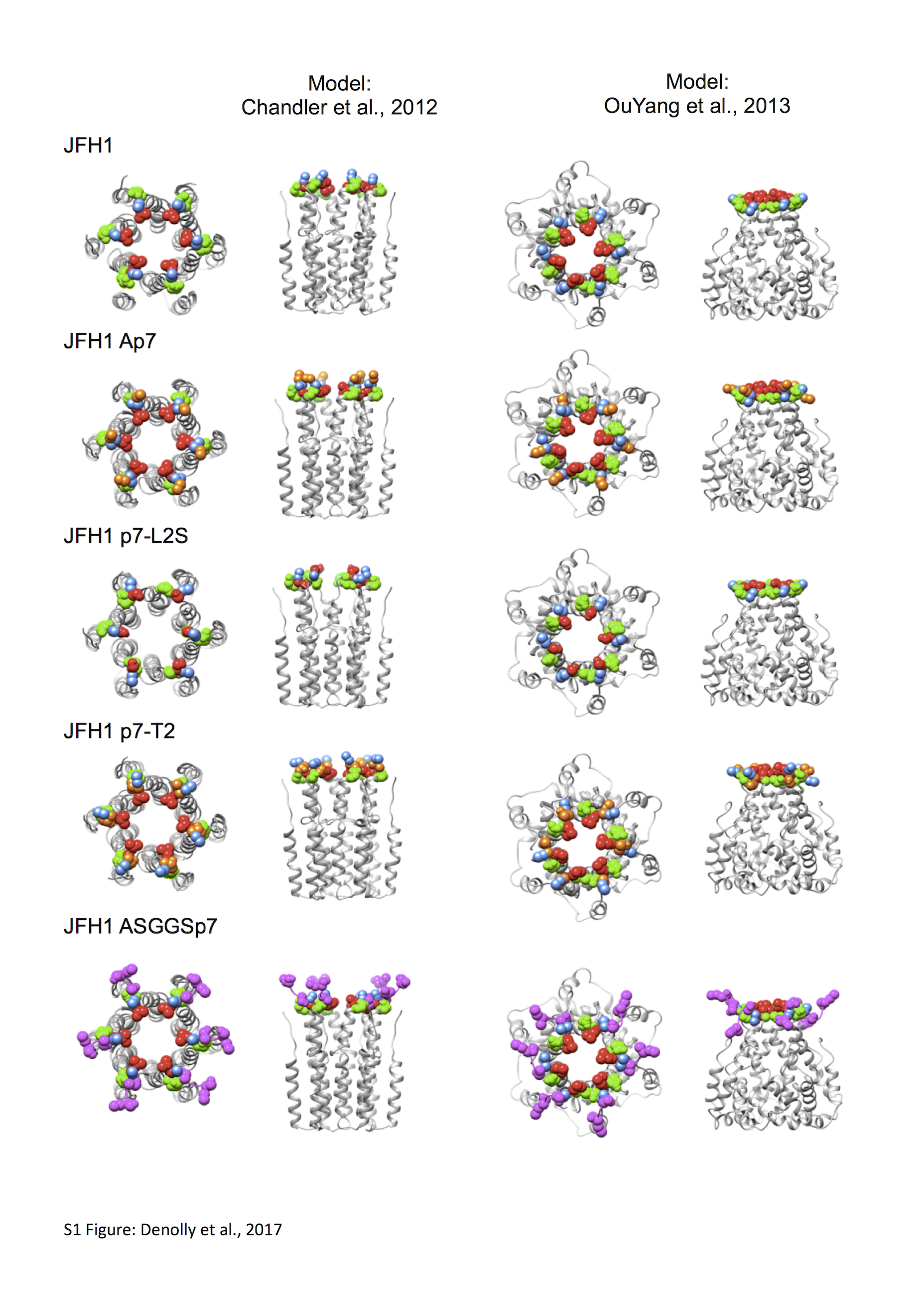

Supplement: S1 Fig — Comparison of three-dimensional homology molecular models of p7 structures using the NMR/MD model in POPC [36] and the NMR model in DPC [35]. The first amino-acid positions are represented in blue, second positions in red, third positions in green. Single amino acid insertions are represented in orange whereas larger insertions are represented in violet. (TIFF) [file ppat.1006774.s001.tiff]

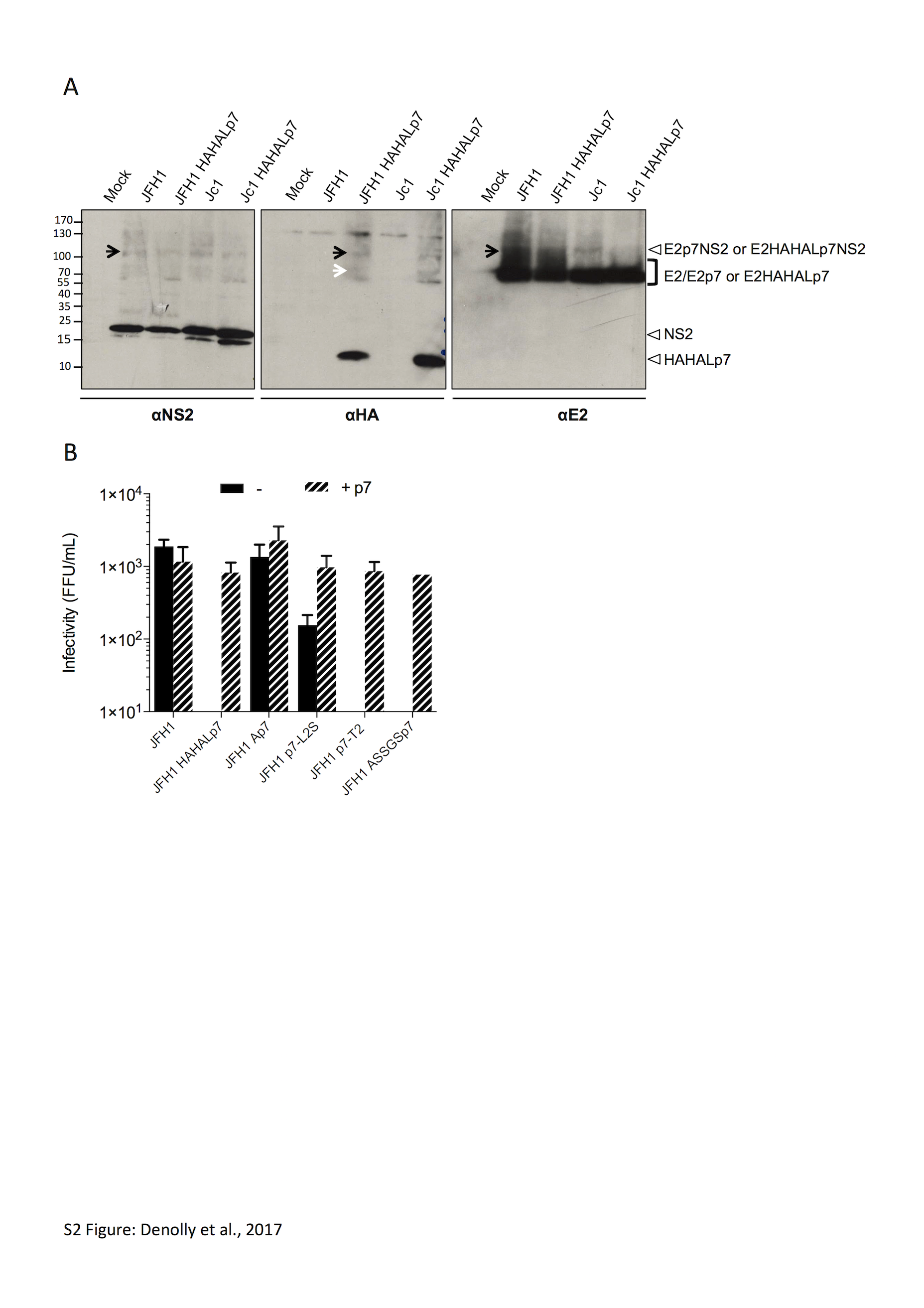

Supplement: S2 Fig — Huh7.5 cells were electroporated with RNAs from parental or p7 ATMI mutant viruses, as indicated. At 72h post-electroporation, expression analyses were performed. (A) NS2, HAHALp7, E2, E2p7 as well as E2HAHALp7 (white arrows) and E2p7NS2 and E2HAHALp7NS2 (black arrows) precursors were revealed using anti-E2, anti-NS2 and HA antibodies, as indicated. (B) The infectivity levels of JFH1-derived p7 ATMI mutant viruses expressed alone (black bars) or with wild-type p7 (hatched bars) are represented. (TIFF) [file ppat.1006774.s002.tiff]

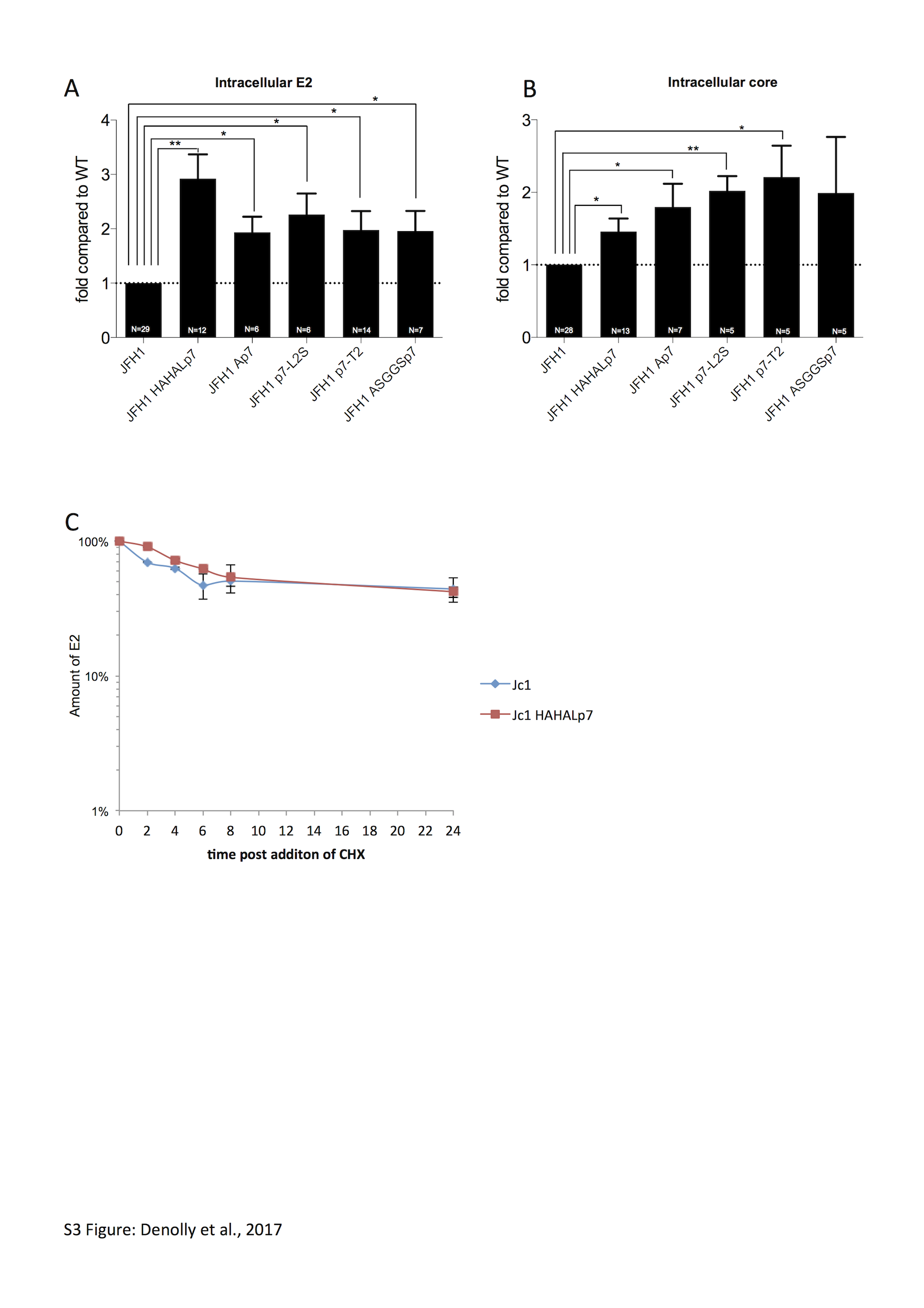

Supplement: S3 Fig — Huh7.5 cells were electroporated with RNAs from parental or p7 ATMI mutant viruses, as indicated. At 72h post-electroporation, expression analyses were performed and determined by quantitative western blot. (A) Levels of intracellular E2 for the JFH1-derived p7 ATMI mutant viruses. (B) Levels of intracellular core for the same mutant viruses. Proteins in (A) and (B) were quantified and normalized after determining the proportion of HCV-positive virus producer cells and the amounts of cellular actin (see Fig 3A). (C) Huh7.5 cells were electroporated with RNAs from parental or Jc1 HAHALp7 mutant virus. At 72h post-electroporation, cells were treated with cycloheximide (100μg/mL) and brefeldin A (1μg/mL). At the indicated time points, cells were counted and the same amounts of cells were lysed. Levels of E2 were determined by quantitative Western blot. The values are displayed relative to expression of E2 and core in JFH1 HCVcc virus-electroporated cells (A, B) or relative to time 0h post-addition of the drugs (C). Data represent mean values ± SEM. The number of experiments performed are indicated below the graphs. (TIFF) [file ppat.1006774.s003.tiff]

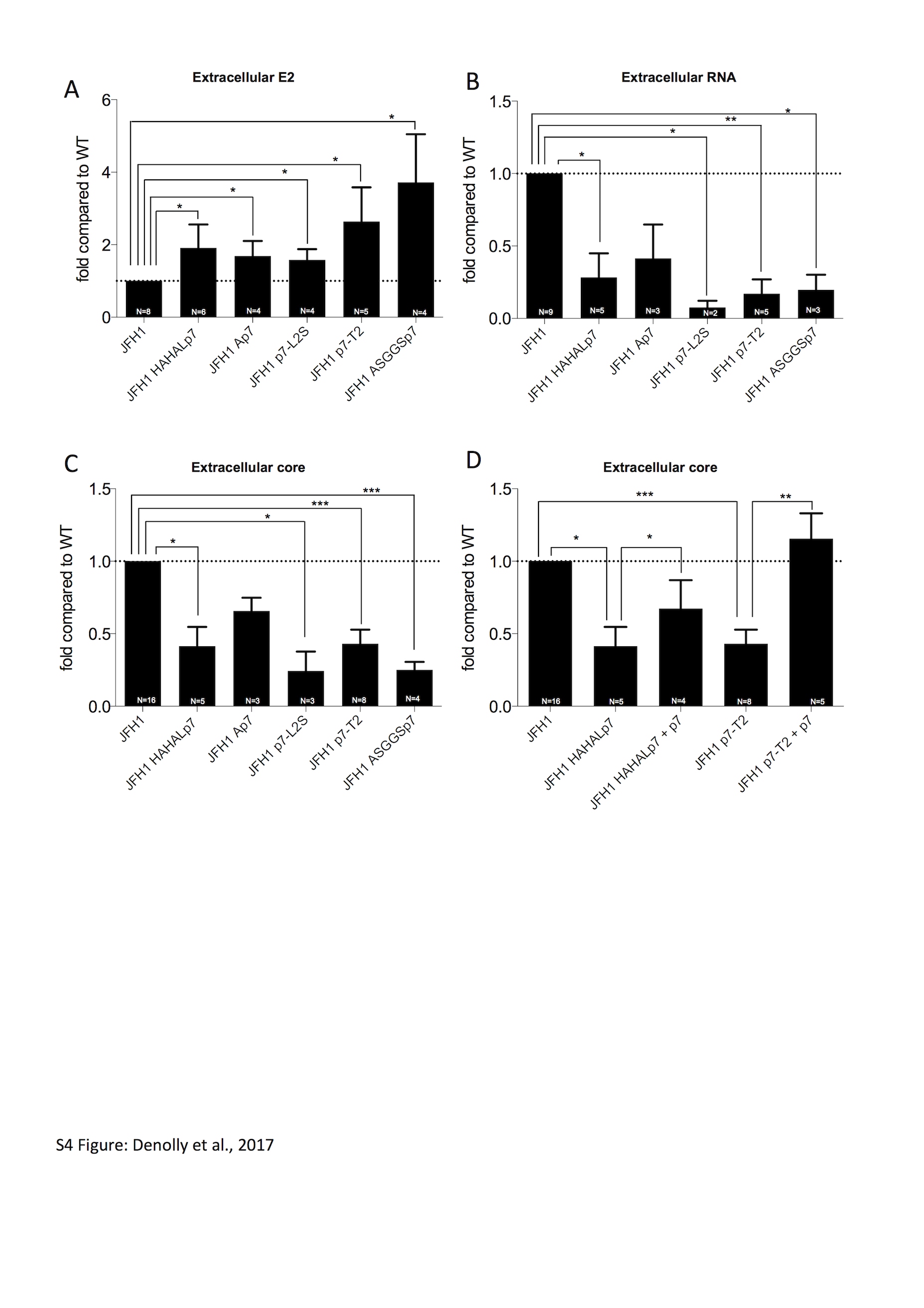

Supplement: S4 Fig — Huh7.5 cells were electroporated with RNAs from parental or JFH1 mutant viruses expressed alone or with wild-type p7. At 72h post-electroporation, analyses were performed and normalized after determining the proportion of HCV-positive virus producer cells (see Fig 3A). (A) Levels of secreted E2 determined by quantitative western blot following GNA lectin pull down of cell supernatants. (B) Levels of secreted HCV RNAs as determined by RT-qPCR. (C, D) Levels of secreted core as determined by CMIA for JFH1 HAHALp7 or JFH1 p7-T2 mutant viruses alone or with WT p7 (D) and for other JFH1-derived p7 ATMI mutants (C). All values are displayed relative to expression of E2, core or RNA values determined in the supernatants of JFH1 virus-electroporated cells (A-C). Data represent mean values ± SEM. The number of experiments performed are indicated below the graphs. (TIFF) [file ppat.1006774.s004.tiff]

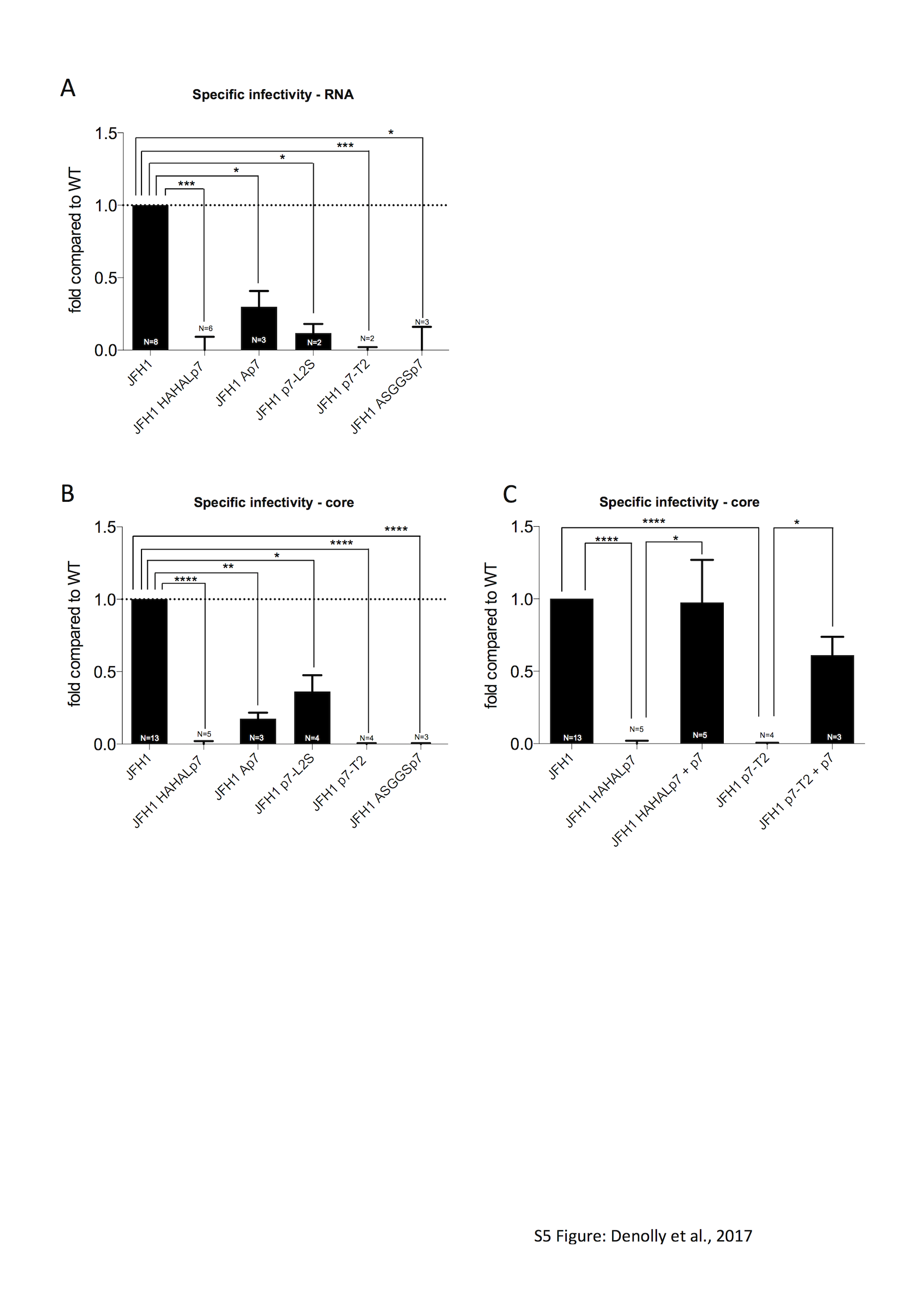

Supplement: S5 Fig — Huh7.5 cells were electroporated with RNAs from parental or JFH1 mutant viruses expressed alone or with wild-type p7. At 72h post-electroporation, infectivity, and RNA and core secretion analyses were performed. (A) Specific infectivity relative to RNA amounts for all JFH1-derived p7 ATMI mutant viruses. (B) Specific infectivity relative to core amounts for all JFH1-derived p7 ATMI mutants. (C) Specific infectivity relative to core amounts for JFH1 HAHALp7 or JFH1 p7-T2 mutant viruses expressed alone or with wild-type p7. Values are displayed relative to expression of specific infectivity in the supernatants of JFH1-electroporated cells. Data represent mean values ± SEM. The number of experiments performed are indicated below the graphs. (TIFF) [file ppat.1006774.s005.tiff]

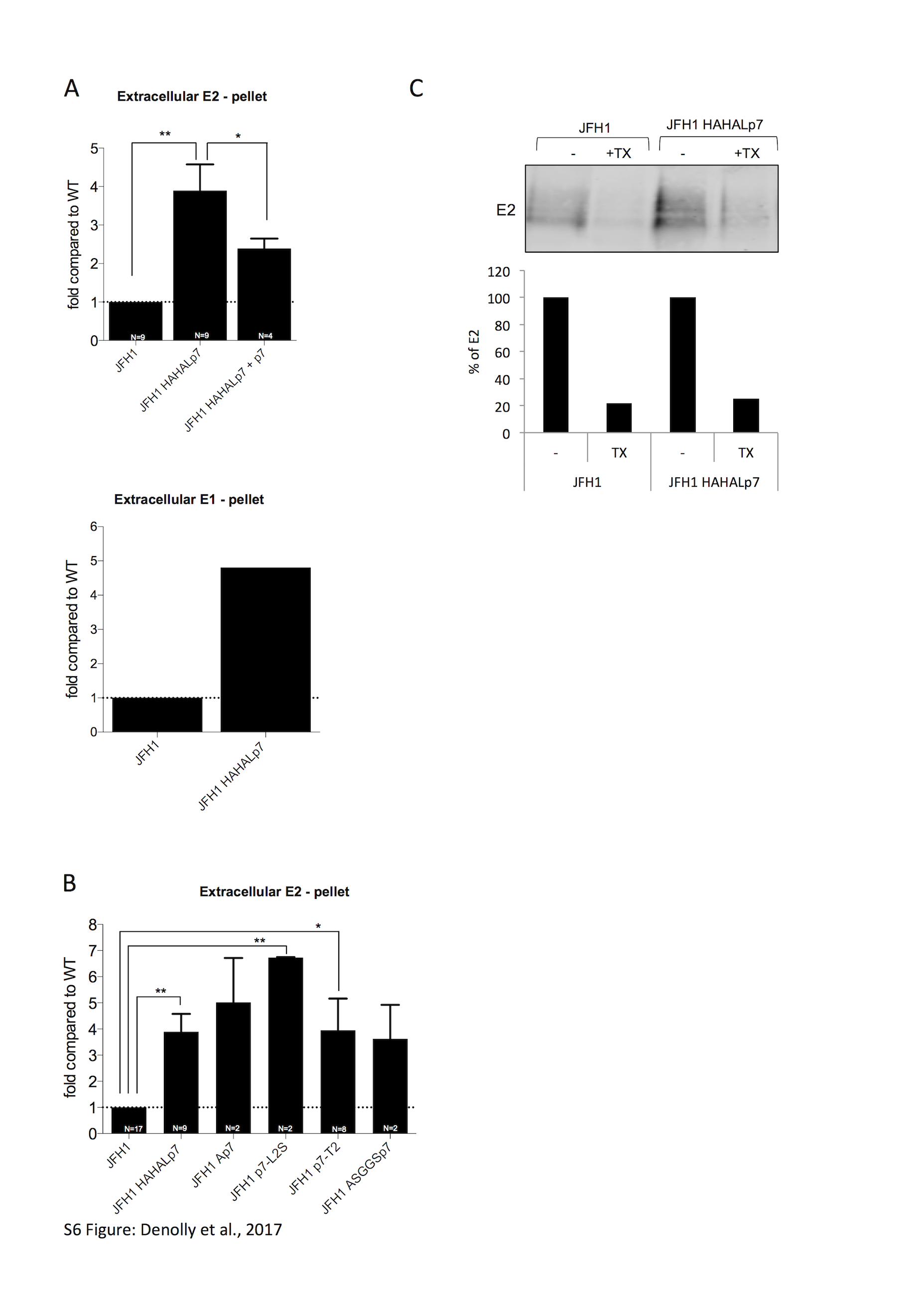

Supplement: S6 Fig — Huh7.5 cells were electroporated with RNAs from parental or JFH1 mutant viruses expressed alone or with wild-type p7. At 72h post-electroporation, quantitative western blot analyses were performed. (A) Level of E2 and E1 in pellet for the JFH1 HAHALp7 mutant virus relative to parental virus. (B) Level of E2 in pellets from ultracentrifuged cell supernatants for all p7 ATMI mutants. (C) Aliquots of supernatant from cells expressing JFH1 or JFH1 HAHALp7 viruses were incubated for 1hr with 1% Triton X-100 or left untreated before ultracentrifugation and analysis of E2 in the pellets by quantitative western blot. Proteins in (A) were quantified and normalized after determining the proportion of HCV-positive virus producer cells. Values are displayed relative to expression of E2 or E1 in the pellets of supernatants from JFH1 virus-electroporated cells. Data represent mean values ± SEM. The number of experiments performed are indicated below the graphs. (TIFF) [file ppat.1006774.s006.tiff]

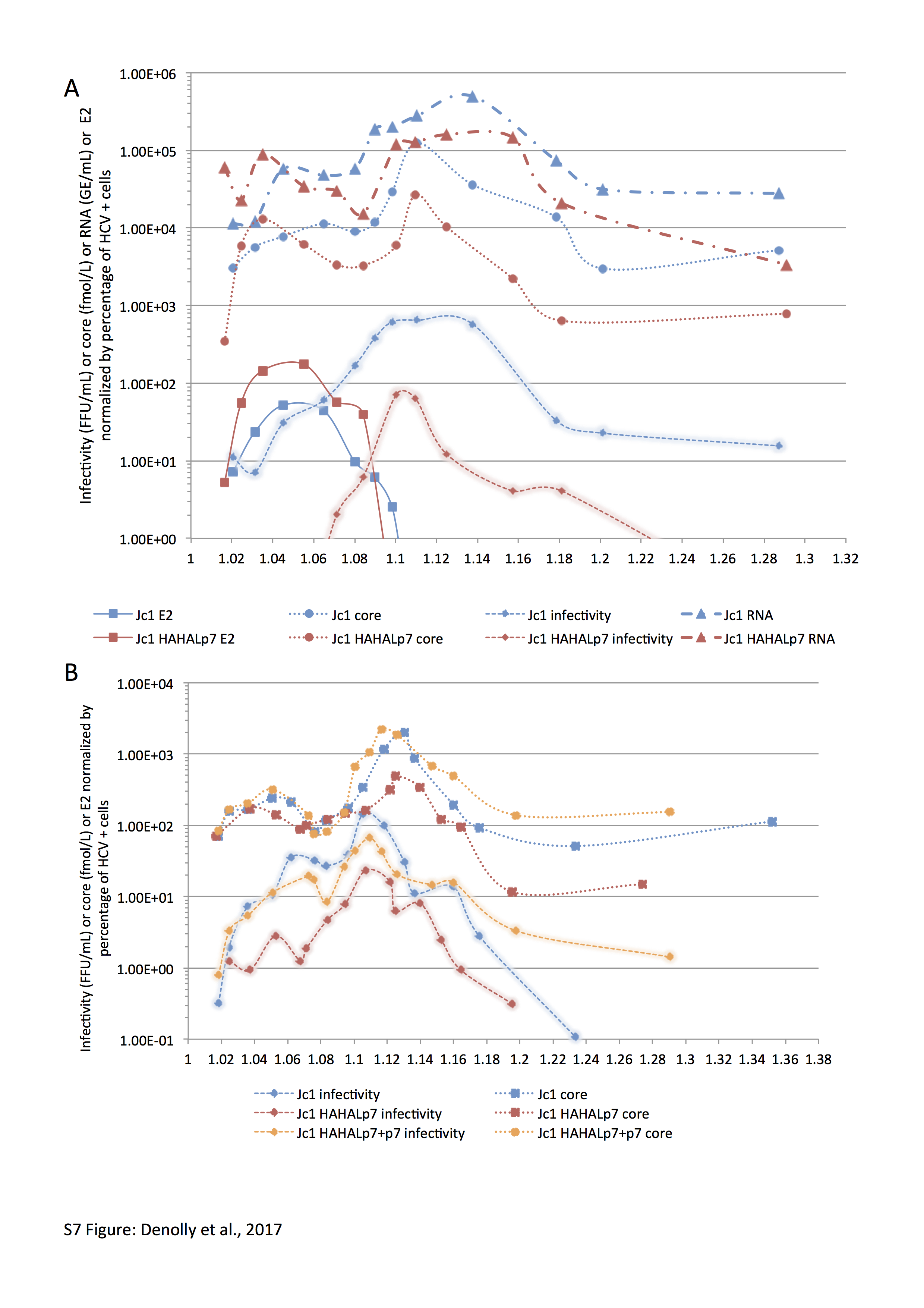

Supplement: S7 Fig — Huh7.5 cells were electroporated with RNAs from parental vs. Jc1 HAHALp7 (A), JFH1 HAHALp7 (C) viruses expressed alone or with wild-type p7 (B). At 72h post-electroporation supernatants were collected and layered on iodixanol buoyant density gradients. (A) Representative gradient profiles of Jc1 and Jc1 HAHALp7 viruses. The levels of E2, core, infectivity, and viral RNAs were quantified and normalized after determining the proportion of HCV-positive virus producer cells (see Fig 3A). (B) Representative gradient profiles of Jc1 and Jc1 HAHALp7 viruses expressed with or without wt p7. The levels of core and infectivity were quantified and normalized after determining the proportion of HCV-positive virus producer cells (see Fig 3A). (C) Representative gradient profiles of JFH1 and JFH1 HAHALp7. The core, E2 and E1 proteins and infectivity were measured in each fraction and expressed of percentages of the sum of fractions. (TIFF) [file ppat.1006774.s007.tiff]

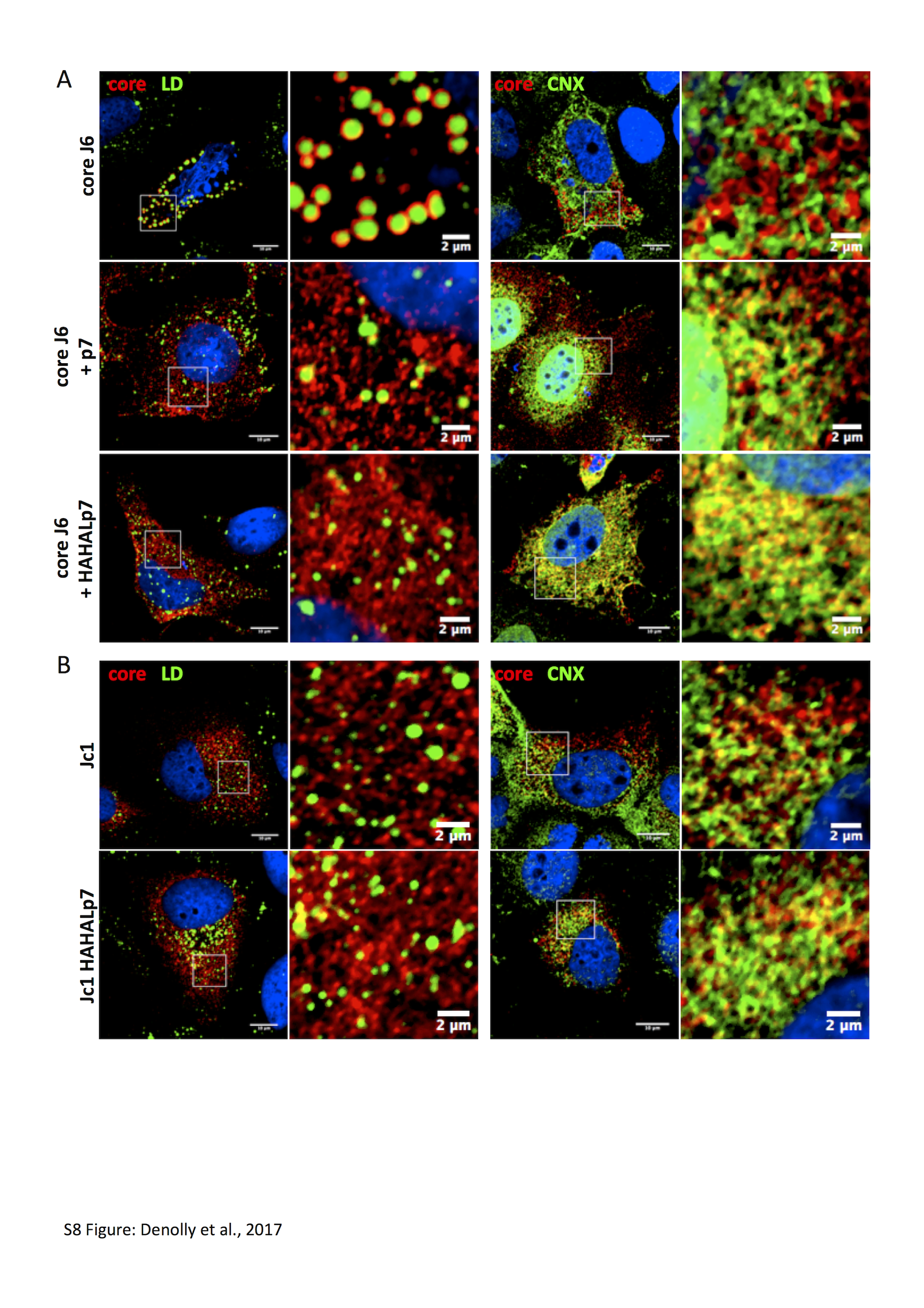

Supplement: S8 Fig — At 72h post-transfection or post-infection, cells were fixed and stained for HCV core (red), LD (green, left panels), calnexin (green, right panels) and nuclei (blue). (A) Confocal microscopy analysis of Huh7.5 cells transfected with constructs expressing J6 core alone or in combination with wt p7 or HAHALp7. (B) Confocal microscopy of Huh7.5 infected with Jc1 or Jc1 HAHALp7 viruses. (TIFF) [file ppat.1006774.s008.tiff]

# A JFH1 HAHALp7 virus

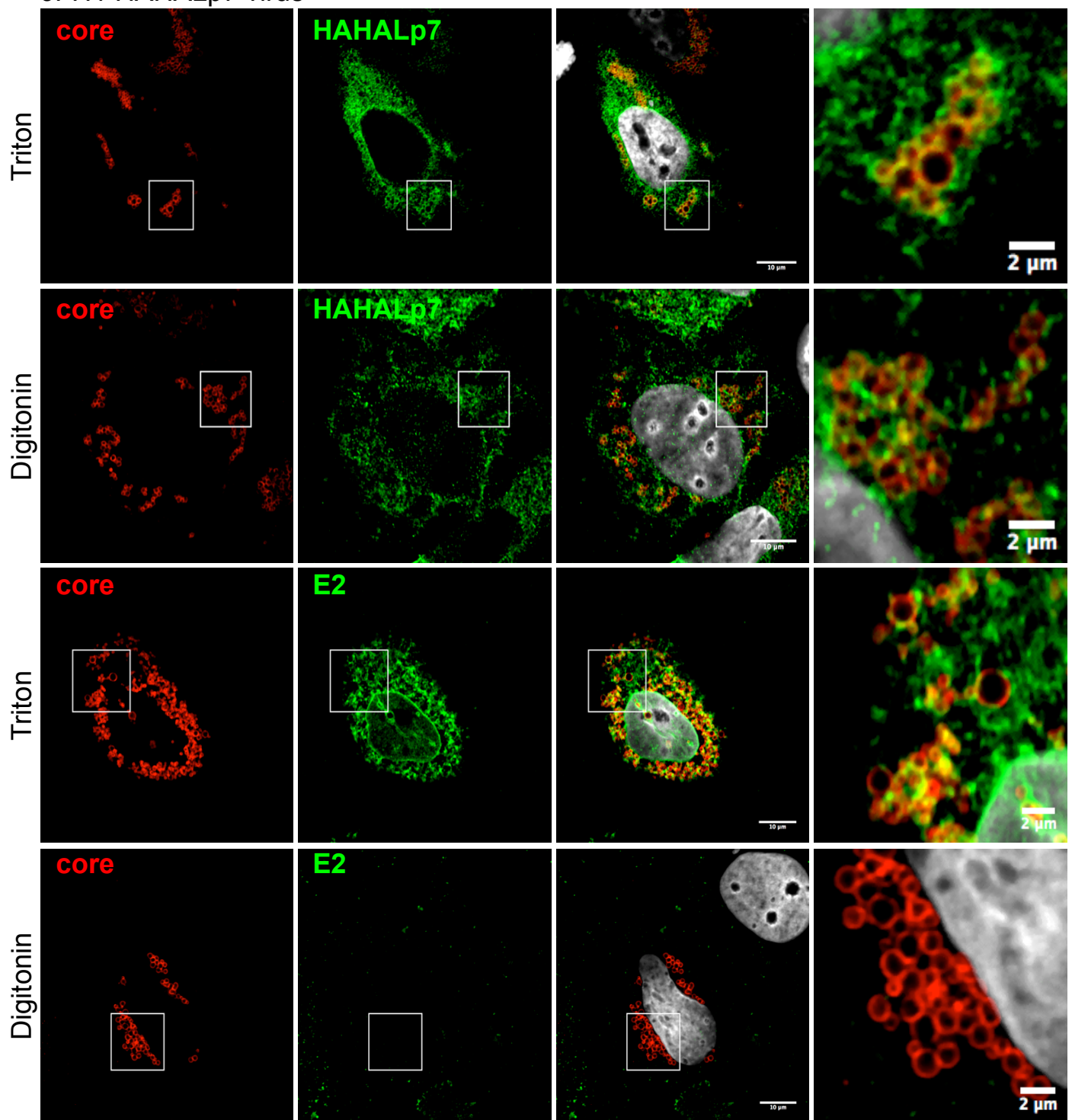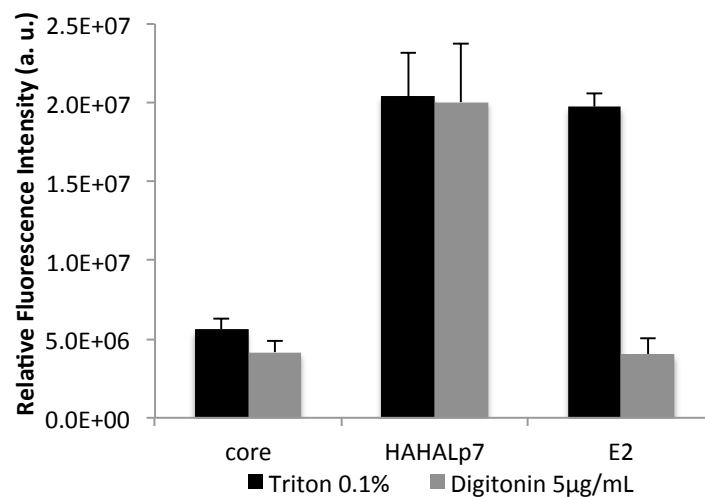

B C-E1E2 + noSPHAHALp7

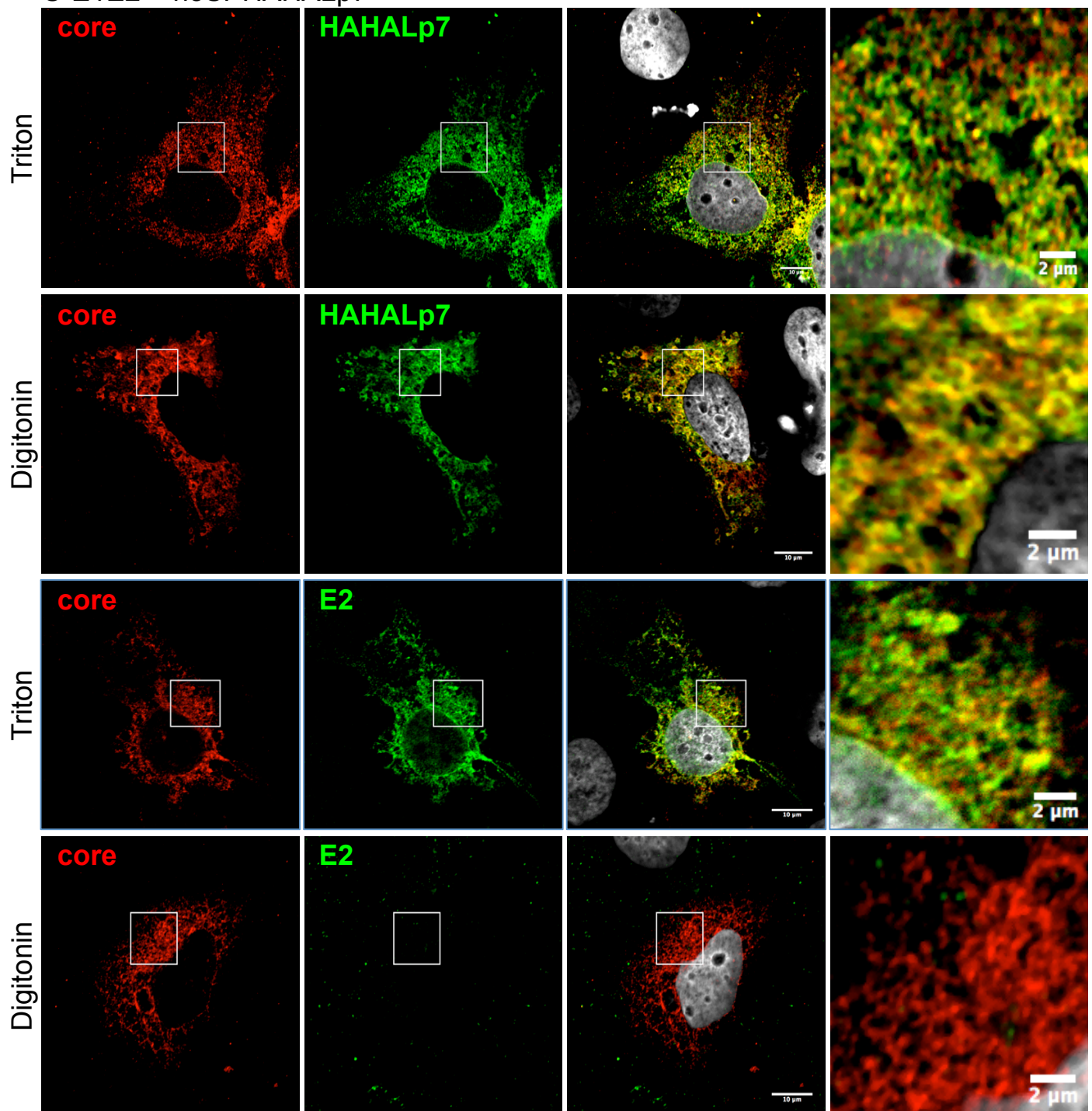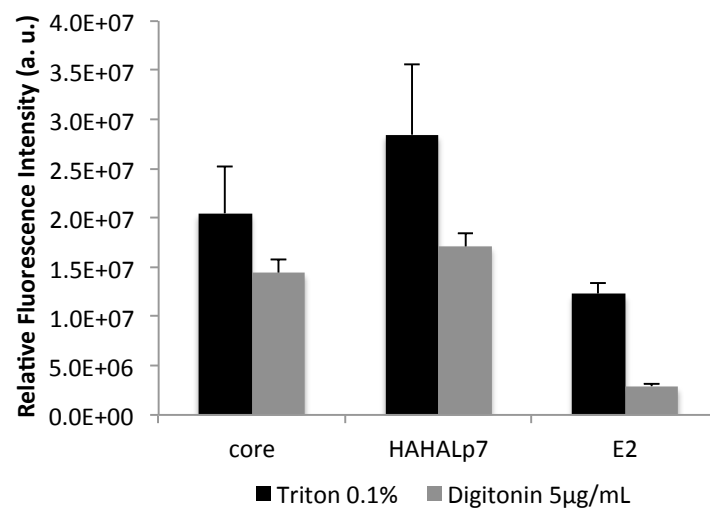

# C C-E1E2 + $\Delta$ E2HAHALp7

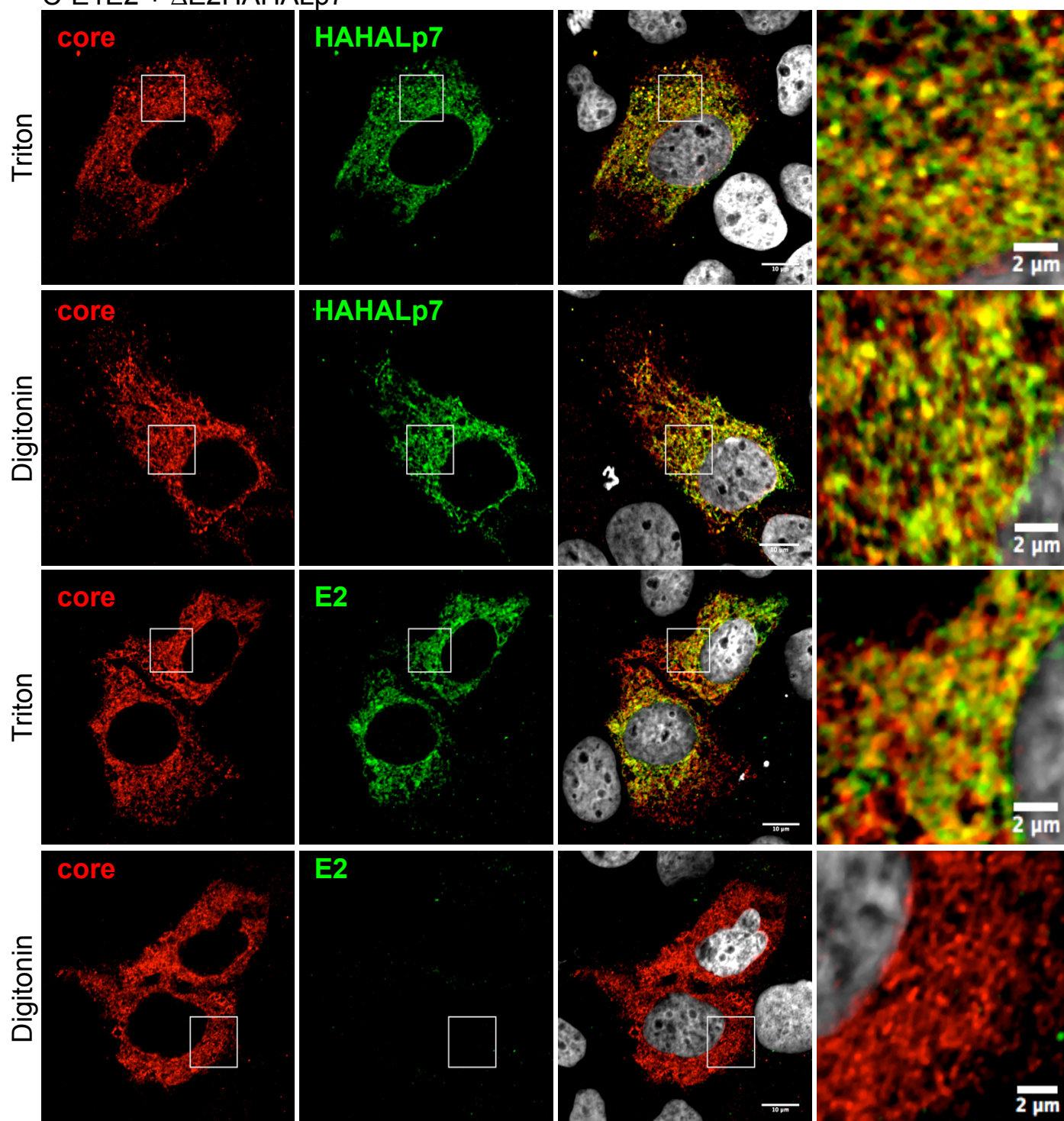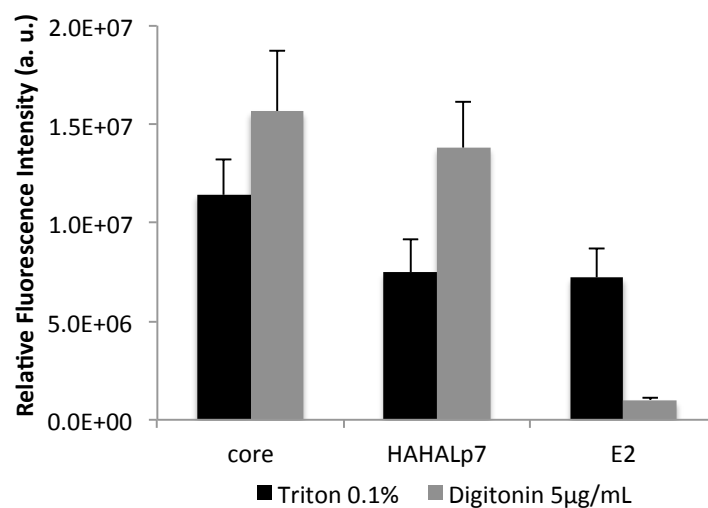

Supplement: S9 Fig — At 72h post-electroporation or transfection, cells were fixed, permeabilized with either Triton X-100 or Digitonin, as indicated, and stained for HCV core (red), HAHALp7 (green), E2 (green) and nuclei (grey). (A) Confocal microscopy analysis of Huh7.5 electroporated with JFH1 HAHALp7 virus RNAs. (B) Confocal microscopy analysis of Huh7.5 cells transfected with core-E1E2 and noSPHAHALp7 expression constructs. (C) Confocal microscopy analysis of Huh7.5 cells transfected with core-E1E2 and ∆E2HAHALp7 expression constructs. The relative fluorescence intensity of each channel was quantified by using ImageJ. (PDF) [file ppat.1006774.s009.pdf]

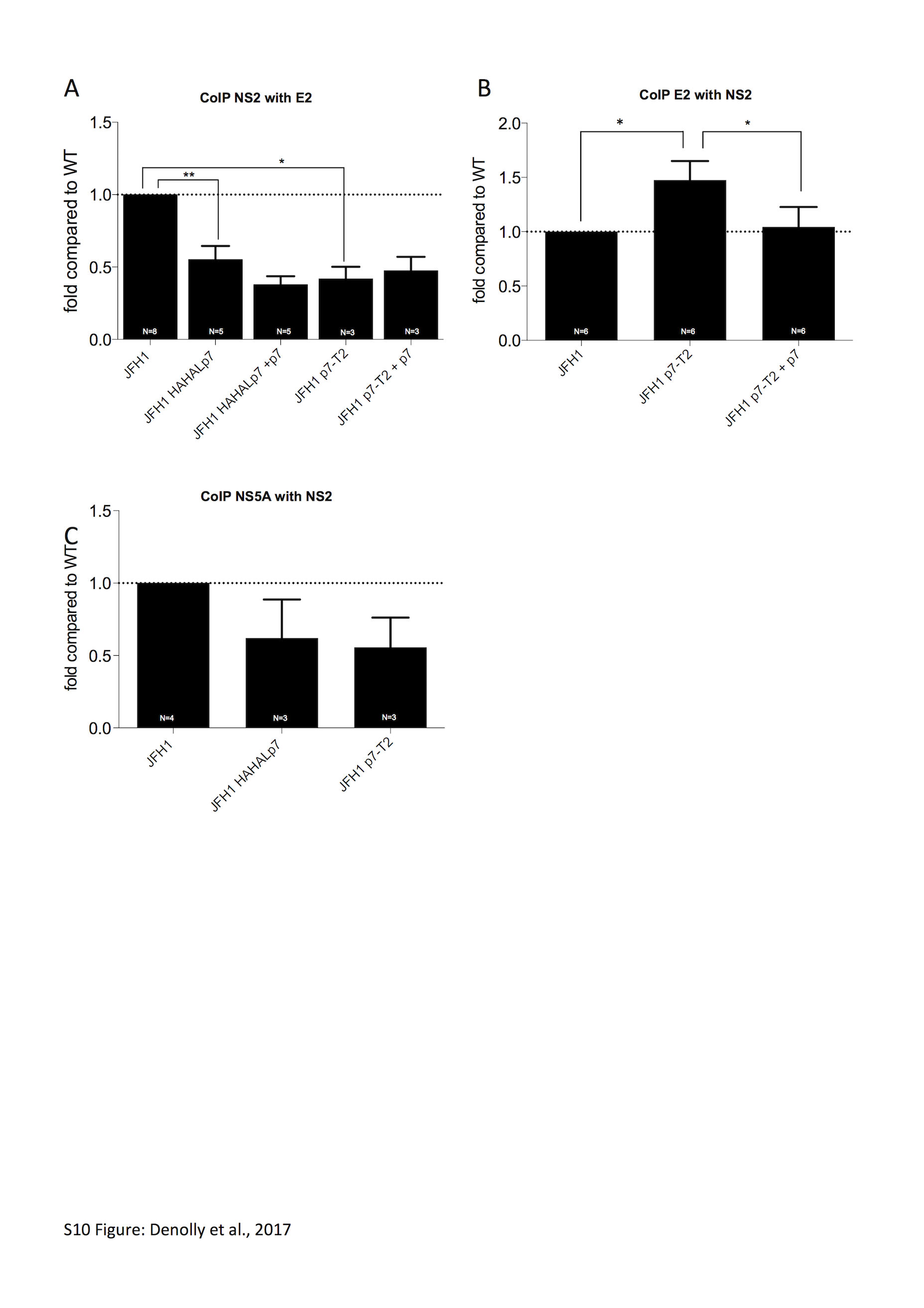

Supplement: S10 Fig — Huh7.5 cells expressing RNAs from parental or JFH1-derived p7 ATMI mutant viruses expressed alone or with wild-type p7 were analyzed at 72h. (A) Levels of NS2 proteins co-immuno-precipitated by E2 antibodies normalized to the amount of immuno-precipitated E2 proteins. (B) Levels of E2 proteins co-immuno-precipitated by NS2 antibodies normalized to the amount of immuno-precipitated E2 proteins. (C) Levels of NS5A proteins co-immuno-precipitated by NS2 antibodies normalized to the amount of immuno-precipitated NS2 proteins. The values are displayed relative to co-immuno-precipitated E2, NS5A or NS2 in JFH1 virus-expressing cells. Data represent mean values ± SEM. The number of experiments performed are indicated below the graphs. (TIFF) [file ppat.1006774.s010.tiff]
